# Translesion DNA synthesis in the context of cancer research

**DOI:** 10.1186/1475-2867-11-39

**Published:** 2011-11-02

**Authors:** Philip A Knobel, Thomas M Marti

**Affiliations:** 1 Laboratory of Molecular Oncology, Clinic and Polyclinic of Oncology, University Hospital Zürich, Häldeliweg 4, CH-8044 Zürich, Switzerland

## Abstract

During cell division, replication of the genomic DNA is performed by high-fidelity DNA polymerases but these error-free enzymes can not synthesize across damaged DNA. Specialized DNA polymerases, so called DNA translesion synthesis polymerases (TLS polymerases), can replicate damaged DNA thereby avoiding replication fork breakdown and subsequent chromosomal instability.

We focus on the involvement of mammalian TLS polymerases in DNA damage tolerance mechanisms. In detail, we review the discovery of TLS polymerases and describe the molecular features of all the mammalian TLS polymerases identified so far. We give a short overview of the mechanisms that regulate the selectivity and activity of TLS polymerases. In addition, we summarize the current knowledge how different types of DNA damage, relevant either for the induction or treatment of cancer, are bypassed by TLS polymerases. Finally, we elucidate the relevance of TLS polymerases in the context of cancer therapy.

## DNA damage response (DDR)

Genomic information is stored as deoxyribonucleic acid (DNA) in every living organism and needs to be protected and maintained to guarantee genomic integrity. Each of the 10^13 ^cells of the human body contains 30'000-40'000 genes encoded by 3 × 10^9 ^base pairs of the DNA [1-3]. The integrity of the DNA is constantly threatened either by spontaneous decay or by damage induced by endogenous and environmental sources. In every single cell, tens of thousands of DNA lesions per day are formed due to spontaneous hydrolysis and the attack of reactive oxygen species (ROS) and other intracellular metabolites [4]. In the context of cancer research, prominent examples for environmental factors which induce DNA damage are ultraviolet (UV)-light inducing [6-4]pyrimidine-pyrimidone photoproducts ([6-4]PP) and cyclobutane pyrimidine dimers (CPDs), and cigarette smoke, which contains a variety of carcinogens, e.g. benzo(α)pyrene (BaP) [5]. Cancer treatment regimens are frequently based on DNA damage inducing agents. For instance, multimodality therapies of solid tumors are often based on cisplatin, a platinum analogue, which induces intra- and interstrand DNA crosslinks [6].

In addition, accurate DNA duplication is an essential step carried out by a complex DNA replication machinery but errors during this process can also compromise genomic integrity. For example, damaged DNA, which cannot be replicated by the high fidelity replicative DNA polymerases, can lead to stalled replication forks and subsequent replication fork breakdown results in chromosomal instability [7].

**Figure 1:**
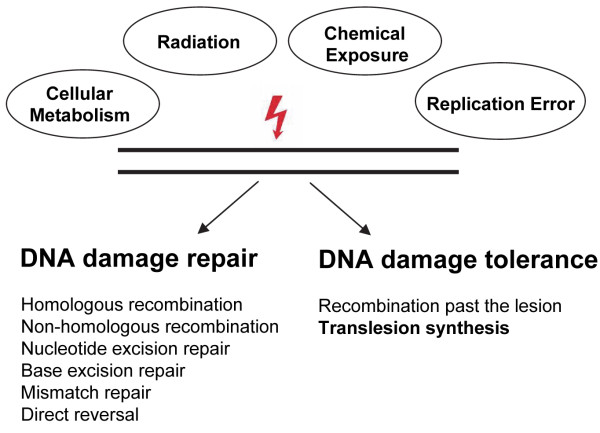
**DNA damage induced by spontaneous decay or endogenous and environmental sources can either be repaired or tolerated (Adapted from **[245]**)**. See text for details.

To counteract the constant loss or the modification of DNA bases, cells evolved a complex and interplaying system, the so-called DNA damage response (DDR) [8,9]. During DDR, DNA lesions are detected, leading to the activation of a signal cascade resulting either in the repair or the tolerance of the DNA damage, thereby regulating the cellular outcome after genomic insult [4] (Figure 1).

The cellular DNA repair machinery includes non-homologous end joining (NHEJ) and homologous recombination (HR) to repair double strand breaks (DSBs), base excision repair (BER) to counteract modification of the nitrogenous bases, nucleotide excision repair (NER) to excise bulky nucleotide alterations such as UV-induced [6-4]PPs, mismatch repair (MMR) to exchange mispaired nucleotides and direct damage repair for reversal of alkylated nucleotides (Figure 1) [10]. Although DNA repair processes are not as accurate as high-fidelity DNA replication, DNA repair is considered to be error-free. In eukaryotes, DNA damage tolerance involves a error-free pathway dependent on homologous recombination and a more mutagenic pathway based on TLS polymerases [11]. In this review we focus on the contribution of TLS polymerases to DNA damage tolerance and their relevance in cancer research.

## Mammalian TLS polymerases: state-of the art

### History and Discovery

In 1956, the group of Arthur Kornberg discovered and described an enzyme purified from *Escherichia coli *(*E. coli*), which is able to create an appropriate copy of its DNA substrate, i.e. DNA polymerase (Pol) I [12]. DNA Pol I was shown to generate a copy of the single-stranded DNA of the small bacterial virus ΦX174. The generated DNA kept the infectious activity, thereby confirming that DNA Pol I is able to generate genetically active DNA [13,14]. DNA Pol II was discovered in 1970 [15,16] and shortly afterwards DNA Pol III was discovered as the third DNA-replicating enzyme [17]. Miroslav Radman and coworkers published in 1974 the "SOS repair" model, proposing that the UV-induced mutations of both λ phage and host *E. coli *are due to a "mutation-prone" cellular replication mechanism [18]. Also in the 1970s, a screen in *Saccharomyces cerevisiae *(S. *cerevisiae*) for reversionless (rev) mutants unable to revert an auxotropic marker after UV irradiation led to the discovery of the first eukaryotic genes encoding error-prone TLS polymerases, i.e. *REV1 *(encoding the TLS Pol Rev1) and *REV3 *(encoding the catalytic subunit of TLS Pol ζ) [19]. A strategy similar to the one which led earlier to the discovery of the UmuDC genes (defects in these genes render cells non-mutable) in *E. coli *[20] resulted in the discovery of and REV7, the structural subunit of TLS Pol ζ, in *S. cerevisiae *[21]. The co-discovery of bacterial and eukaryotic TLS polymerases revealed the conservation of a cellular process that had until then been considered to be a bacterial-specific function. Subsequently, it was shown that DNA Pol II is part of the "SOS repair" [22,23]. Although the DinB [24] and the UmuDC [20] genes of *E. coli *were discovered much earlier, it was shown only in the 1990s that these gene products constitute the error-prone DNA Pol IV [25] and Pol V, respectively [26,27]. DNA Pol IV and Pol V are inducible by DNA damage and can efficiently bypass various forms of DNA lesions thereby generating most of the SOS-repair dependent mutations [28]. Other mammalian TLS polymerases such as Pol η (eta; *hRAD30A/XPV*), Pol ι (iota; *hRAD30B*), Pol κ (kappa; *DINB1*) and Pol θ (theta; *POLQ*) were identified by searches for homologues of genes of previously identified TLS polymerases [25,29-32]. The TLS Pol μ (mu) [33,34] and Pol λ (lambda) were discovered and described more recently [33,35]. The most recently described TLS Pol ν (nu) was found due to homology with *mus308 *[36].

The ability of eukaryotic TLS polymerases to bypass DNA lesions, was firstly described for the yeast TLS Pol ζ, mediating the bypass of UV-induced thymine-thymine cyclobutane pyrimidine dimers (TT-CPDs) [37]. The property of Rev1 to insert deoxycytidine monophosphate (dCMPs) opposite abasic sites was first described in yeast [38]. Subsequently, the role of human TLS Pol ζ to bypass DNA lesions [39] and the function of human Rev1 as dCMP transferase opposite abasic sites [40] were proposed. The UV lesion bypass activity of human TLS Pol η was discovered by the fact that xeroderma pigmentosum variant (XPV) patients show increased susceptibility to UV-induced skin cancer [41] and hypermutability [42] due to a defect of TLS Pol η. The human TLS Pol ι was shown to be able to incorporate deoxynucleotides opposite the 3' T of [6-4]PPs and abasic sites [43] and opposite N2-adducted guanines [44]. Human TLS Pol κ was identified and it was first shown that TLS Pol κ protect cells against the lethal and mutagenic effects of BaP [45]. Human TLS Pol θ was identified more recently and it was shown that TLS Pol θ is implicated in somatic hypermutation (SHM) [46-48]. Similarly, TLS Pol λ and TLS Pol μ are both implicated in V(d)J recombination during the immunoglobulin (IgG) diversification process [49,50] whereas TLS Pol ν is able to bypass thymine glycols (Tg) [51]. It can not be excluded that additional TLS polymerases will be discovered in mammalian genomes.

### Fidelity of TLS

TLS is defined as the incorporation of a nucleotide across DNA damage followed by extension of the potentially mispaired primer-template, which can be error-free or error-prone [52]. The basic necessity for the presence of TLS polymerases reflects a trade-off between the maintenance of genomic integrity by avoiding replication fork breakdown and subsequent chromosomal instability and the occurrence of mutations on the nucleotide level by the TLS polymerases mediated DNA damage bypass reaction.

Although the tertiary structure consisting of palm, thumb and fingers is conserved among the different polymerase families, the thumb and fingers of the TLS polymerases are smaller. Compared to the DNA replication polymerases where the fingers tightly bind the incoming dNTPs and make a conformational change upon correct Watson-Crick base pairing, the active site of TLS polymerases is more open and less constrained to reject wrong paired base pairs. Therefore, TLS polymerases are able to mediate the bypass reaction of non-coding DNA lesions. The additional little finger of the Y family TLS polymerases supports the stabilization of the template DNA and influences fidelity and activity [53].

The error rate of DNA replication polymerases of the families A, B and C including correct incorporation of the nucleotide and the proofreading activity is between 10^-6 ^and 10^-8^. Auxiliary proteins such as proliferating cell nuclear antigen (PCNA) and replication protein A (RP-A) [54] and postreplicative MMR decrease the error rate to 10^-8 ^and 10^-10 ^. The error rate of the TLS polymerases ranges from 10^-1 ^to 10^-3 ^for replication of undamaged DNA [55,56]. Due to the characteristic low fidelity DNA synthesis and the lack of an exonuclease proofreading activity, it was initially assumed that TLS is generally a mutagenic process. Recently it became clear that the use of specialized TLS polymerases at specific lesions can be error-free. The best example is the ability of TLS Pol η to bypass TT-CPDs, the main DNA lesion induced by both UVB and UVA radiations, in a non-mutagenic manner [57,58].

In this context, it is important to address whether the accurate bypass of a particular lesion by a TLS Pol *in vitro *can be used as an indicator whether this polymerase also processes the corresponding lesion *in vivo*. In addition to the TT-CPDs *in vitro *bypass activity of TLS Pol η, it was also shown that inactivation and deletion of Pol η decreases UV survival of human [59,60] and yeast cells [29,61]. Similarly, TLS Pol θ is able to bypass oxidative DNA lesions, i.e. apurinic/apyrimidinic (AP) sites *in vitro *[62] and knockout of TLS Pol θ in the chicken DT40 B-cell line resulted in hypersensitivity to hydrogen peroxide (H_2_O_2_) [63]. Thus, in general, the *in vivo *sensitivity to a DNA damage inducing agent of cells deficient for a specific polymerase can be predicted by the *in vitro *ability of the Pol to bypass the induced DNA lesion. However, it became clear that the bypass reaction of most DNA adducts requires the concerted action of protein complexes containing several TLS polymerases. Inactivation or deletion of a TLS polymerase can disrupt protein-protein interactions essential for lesion bypass therefore indirectly affecting the *in vivo *sensitivity to the DNA lesion inducing agent. Thus, based on the *in vivo *sensitivity of an inactivation or deletion mutant, it can not be concluded that the induced DNA damage is processed by the modified TLS Pol activity. For example, although TLS Pol ζ is sensitive to UV-irradiation, other TLS polymerases perform the bypass reaction whereas Pol ζ mainly performs the subsequent extension step [64] (see also Table 1).

**Table 1:** TLS opposite DNA lesions by mammalian one and two-polymerase mechanisms

DNA lesion	Insertion	Extension	Outcome
Apurinic/apyrimidinic (AP) site	TLS Pol β/κ/θ/η/λ/μ/δ+PCNA	same Pol	Mutagenic [184,189,62,186,191] [187,188,190,191]
	REV1	(Pol η; Pol δ/PCNA)	Mutagenic [191]
	TLS Pol ι	(Pol η; Pol δ/PCNA)	Mutagenic [191]

7, 8-dihydro-8-oxoguanine (8-oxo-G)	TLS Pol ι/κ/μ	same Pol	Accurate [197]/Mutagenic [196,190]
	TLS Pol β/λ/η	ND	Mutagenic/Accurate (+PCNA and RPA) [198,199]

Thymine glycol (Tg)	TLS Pol κ	TLS Pol ζ	Accurate [201]
	TLS Pol ν	same Pol	Accurate (5S-Tg); Mutagenic (5R-Tg) [51]
	TLS Pol β/λ	same Pol	Mutagenic [200]
	TLS Pol θ	ND	Mutagenic [62]

[6-4]photoproduct ([6-4]PP)	ND	TLS Pol ζ	Accurate? [205]
	TLS Pol ι	TLS Pol θ	Mutagenic [70,205]
	TLS Pol η	ND	Mutagenic [51,205]

Cyclobutane pyrimidine dimer (CPD)	TLS Pol η	same Pol	Accurate [51,208,209]
	TLS Pol μ	TLS Pol ζ	Accurate [190]
	TLS Pol ι	TLS Pol ζ	Mutagenic [160]
	TLS Pol κ	TLS Pol ζ	Mutagenic [160]
	ND	TLS Pol ζ	Mutagenic [160]

Benzo[α]pyrene-guanine (BP-G)	TLS Pol κ/η/ND	TLS Pol ζ	Accurate/Mutagenic/Accurate? [64]
	TLS Pol κ/μ	same Pol	Mutagenic [190,211]

Intrastrand-crosslink	TLS Pol η	same Pol; Pol ζ/REV1	Accurate [186,213,64,105]
	Pol β/ζ/μ	same Pol	Mutagenic [122,212,64,214,215]
	TLS Pol κ	TLS Pol ζ	Mutagenic [64]

Interstrand-crosslink (ICL)	Recombination-independent ICL repair including NER, REV1 and TLS Pol ζ		Mutagenic [221]
	Recombination-dependent ICL repair including NER, REV1, TLS Pol ν and ζ		Mutagenic [76,104,222]

Abbreviations: One-polymerase mechanism (same Pol); not determined (ND). Note: TLS activity by a one-polymerase mechanism was in general determined by *in vitro *experiments. Therefore, it can not be excluded that the extension step *in vivo *is performed by another TLS polymerase, i.e. a two-polymerase mechanism.

### TLS polymerase families

**Figure 2:**
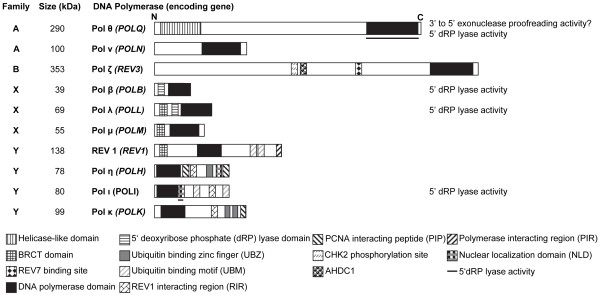
**Overview of TLS polymerases (Adapted from **[246]**)**. See text for details.

The Y-family TLS polymerases η, ι, κ and REV1 [65] and the B-family TLS Pol ζ [66,67] perform most of the TLS in mammalian cells and are well characterized. Less is known about the member of the A-family TLS polymerases, which were more recently identified. An involvement in DNA damage tolerance was also shown for the TLS polymerase members of the X-family although their involvement in DNA repair processes might be their main cellular function (Figure 2).

### Family A: TLS polymerases theta (θ) and nu (ν)

The A family polymerases consist of DNA Pol γ (gamma), which is the mitochondrial DNA replicase and the TLS Pol θ and TLS Pol ν.

### TLS Pol θ

The *POLQ *gene encoding TLS Pol θ was mapped on chromosome 3q. The C-terminal region of TLS Pol θ includes the polymerase motifs A, B and C, which are typical for A family polymerases whereas the N-terminal region contains an ATP domain. The *POLQ *gene encodes a protein of 2592 amino acids (aa). The protein sequence shares homology to the Mus308 protein of *Drosphila melanogaster *[30,68]. Further research estimated a protein of 290 kDa, with an N-terminal ATPase helicase domain although no helicase activity could be detected so far [68]. Additionally, TLS Pol θ has been shown to have 5' dRP lyase activity that is involved in short patch BER *in vitro *[69]. It was shown that TLS Pol θ is able to bypass AP sites and also thymine glycols, e.g. a DNA damage product of ionizing radiation and other oxidative mutagens. TLS Pol θ preferentially incorporates adenine (A) opposite an AP site, which allows DNA replication to continue by using the incorporated nucleotide as a primer (henceforth referred to as extension step) [62]. Additionally, TLS Pol θ can carry out the extension step from mismatches after error prone dNTP incorporation by human TLS Pol ι [70] or *S. cerevisiae *TLS Pol ζ opposite [6-4]PPs *in vitro *[43]. Due to the lacking 3' to 5' exonuclease proofreading activity [68], the fidelity of TLS Pol θ during dNTP incorporation is lower than usual for A family polymerases [62]. It is proposed that TLS Pol θ has a function in SHM of IgG diversification due to misincorporation of nucleotides opposite AP sites and the low fidelity during DNA replication of undamaged DNA [46-48]. Conversely, it was suggested that human TLS Pol θ from HeLa cells nuclear extracts synthesizes DNA with a high fidelity and possesses 3' to 5' exonuclease proofreading activity [71].

TLS Pol θ mutant mice show an increase of spontaneous and radiation-induced micronuclei formation [72,73] and TLS Pol θ knockout chicken DT40 B-cell line shows hypersensitivity to hydrogen peroxide (H_2_O_2_) [63]. Furthermore, CH12 mouse B lymphoma cells containing a knockdown of TLS Pol θ showed elevated sensitivity to UV irradiation, the crosslinking agent mitomycin C and cisplatin, etoposide, ionizing irradiation and the alkylating agent methyl methanesulphonate (MMS) [74]. Thus, TLS Pol θ is involved in the tolerance of a broad range of DNA adducts, which indicates that TLS Pol θ mainly functions as an extender polymerase similar to TLS Pol ζ.

### TLS Pol ν

The full length *POLN *gene comprises 24 exons with a length of 900 aa. The *POLN *gene is located on chromosome 4p16.2 and is deleted in approximately 50% of breast carcinomas [75]. The *POLN *gene encodes a protein with a size of 160 kDa. The C-terminal polymerase domain of TLS Pol ν consists of the typical A family polymerase motifs A, B and C and shares 29% identity with the C-terminus of TLS Pol θ. Neither a 3' to 5' nor a 5' to 3' nuclease domain were identified [36]. *In vitro *experiments showed the ability of TLS Pol ν to bypass thymine glycols [51]. Interestingly, it has been shown that TLS Pol ν is involved in cross link repair and homologous recombination. In detail, depletion of TLS Pol ν sensitizes HeLa cells to the DNA cross-linking agent mitomycin C but not to UV irradiation [76]. Depletion of TLS Pol ν in U2OS cells reduced the efficiency of homologous recombination in a GFP-based reporter assay and increased the sensitivity of HeLa cells to camptothecin-induced DSBs, i.e. a substrate for homologous recombination [77]. Contradictorily, deletion of POLN did not sensitize chicken DT40 cells to campothecin. However, the same study showed that TLS Pol ν in chicken DT40 cells has a dominant role in homologous recombination-dependent immunoglobulin gene conversion and in TLS-dependent immunoglobulin hypermutation [78].

### Family B: TLS Pol zeta (ζ)

The family B includes the highly accurate DNA polymerases δ (delta), ε (epsilon), α (alpha), and the error-prone TLS Pol ζ [79,80].

### TLS Pol ζ

Unlike the replicative DNA polymerases δ and ε, TLS Pol ζ lacks the 3' to 5' exonuclease proofreading activity. The human TLS Pol ζ and its yeast homologue are heterodimeric proteins consisting of the catalytic subunit REV3 and the structural subunit REV7 [37,81]. The human REV3 protein has two transcripts that have a length of 3052 and 3130 aa and the larger protein has a size of 353 kDa compared to 173 kDa of the yeast REV3. The discrepancy in size between the yeast REV3 and the human REV3 is due to the exon 13 with a length of 1388 aa. Human REV3 shows ~36% identity with the N-terminal region, ~29% identity with the central REV7 binding region and ~39% identity with the C-terminal DNA polymerase region of the yeast homologue. The C-terminal region consists of six B-family conserved DNA polymerase motifs and two zinc finger motifs [66,67,82]. Human *REV3 *is located on chromosome 6q21 and its mouse equivalent on chromosome 10 [83,84]. Interestingly, the *REV3 *gene is located in the chromosomal region 6q21 within the fragile site FRA6F, which is known to be commonly deleted in several types of human leukemias and solid tumors [85]. The human *REV3 *contains an out-of-frame ATG in the 5' region that reduces the rate of correct transcripts. Moreover, a sequence upstream of the AUG initiator codon has the potential to form a stem-loop hairpin that lowers the rate of translation. It is suggested that the characteristic structural features in combination with the alternative splicing are responsible for the observed low *REV3 *expression levels [66,83]. Indeed, the protein concentration of REV3 in *Xenopus laevis *egg extracts is much lower than those of other replication and repair proteins and does not change within the early embryonic development [86].

In contrast to viable *REV3 *null yeast mutants, the disruption of *REV3 *in mice causes embryonic lethality around midgestation [87-90]. It is known that during the early stages of embryogenesis checkpoints are actively silenced [91] to allow rapid cell division and it was proposed that REV3 is essential during this strict temporal program. The embryonic lethal effect could not be rescued by the absence of p53 suggesting a p53-independent pathway. However, mouse embryonic fibroblasts (MEFs) with a p53-deficient background could be generated [92,93].

An *in vitro *study showed that *REV3 *expression levels are directly regulated by a p53 responding element in the *REV3 *promoter region. In addition, *REV3 *expression was increased after DNA damage induction in a p53-dependent manner [94]. An independent study also showed increased *REV3 *mRNA level after cisplatin treatment [95]. Over-expression of *REV3 *in yeast led to an elevated rate of UV-induced mutagenesis [96]. These findings together with the embryonic lethal effect after *REV3 *abrogation indicate that the level of REV3 has to be tightly regulated to maintain genomic integrity.

Within the exon 13 of *REV3*, serine 995 was shown to be phosphorylated by CHK2 [97]. In addition, REV3 shares an AT-hook domain with AHDC1, which has been proposed to be phosphorylated by either ATM and ATR upon DNA damage, indicating a putative regulation of REV3 by ATM and ATR [98].

The human TLS Pol ζ is thought to be the major contributor to the error-prone bypass of DNA lesions. Relevant for tumorigenesis, *REV3 *knockout in MEFs leads to increased chromosomal instability in a p53-deficient background [92]. Moreover, in mice with a conditional deletion of REV3, thymic lymphomas occurred with decreased latency and elevated incident in a p53-deficient background [99]. Relevant for cancer therapy, REV3 down-regulation in human foreskin fibroblasts revealed decreased mutation frequency after treatment with UV or BaP-diolepoxide [100]. Similarly, MEFs derived from mice expressing *REV3 *antisense revealed decreased mutagenic frequency after UV treatment [101]. Recent *in vitro *and *in vivo *studies revealed that inhibition of *REV3 *expression increased the sensitivity of lymphoma to cisplatin [102]. Similarly, REV3 depletion in combination with cisplatin treatment decreased the growth rate of a p53-deficient non small cell lung cancer cell line (NSCLC) transplanted into mice and prolonged the survival of the host. The same study showed that the frequency of 6-thioguanine resistant colonies after cisplatin treatment was reduced in REV3 deficient cells compared to the control [103]. Thus, although REV3 is involved in the maintenance of genomic integrity, inhibition of REV3 expression might enhance the anti-tumor activity of DNA-damage inducing agents as discussed below.

Recent findings propose TLS Pol ζ to have a function not only in TLS synthesis but also in DNA repair, e.g. *REV3 *deletion impairs HR [95] and ICL repair [104,105]. Furthermore, SHM and/or class switch recombination (CSR) of IgG were affected by TLS Pol ζ ablation [106].

Although human REV3 contains a REV7 binding region no interaction between full-length REV3 and REV7 could be demonstrated so far. However, it was shown, that a human REV3 fragment interacts with full-length REV7 and a part of human REV7 interacts with human REV3 and REV1 [107]. The human REV7 protein has a length of 211 aa and a size of 24 kDa and shares ~23% identity with the yeast REV7. The human *REV7 *is located on the human chromosome 1p36. REV7 displays ~23% identity with the spindle checkpoint assembly protein MAD2 and it is therefore also known as MAD2B and MAD2L2 in higher eukaryotes. The REV7 contains a HORMA (Hop1/Rev7/Mad2) domain that is known to interact with chromatin [108]. Additionally, REV7 interacts with CDH1 and CDC20 of the anaphase-promoting complex/cyclosome (APC/C) [109] and the protein MAD2, a spindle checkpoint protein [81], indicating that REV7 is involved in the regulation of mitosis. Interestingly, the bacterial pathogen *Shigella *delivers the effector IpaB into epithelial cells to efficiently colonize the epithelium. It has been shown that IpaB interacts with MAD2L thereby inducing a cell cycle arrest [110].

### Family X: DNA Pol beta (β), TLS Pol lambda (λ) and TLS Pol mu (μ)

The polymerases of the X family include DNA Pol β, terminal deoxynucleotidyl transferase (TdT), TLS Pol λ and TLS Pol μ. All the X family polymerases lack the 3' to 5' exonuclease proofreading activity.

### DNA Pol β

DNA Pol β is a 39 kDa monomeric protein and the encoding gene *POLB *is located on chromosome 8 in both mice and human [111]. DNA Pol β consists of two protease resistant segments linked by a short protease sensitive segment indicating that DNA Pol β activity might be controlled by proteolytic activity. The 8 kDa N-terminal lyase domain shows a strong affinity to ssDNA [112], whereas the 31 kDa C-terminal polymerase domain specifically binds double-stranded nucleic acids [113]. The 31 kDa polymerase domain consists of three subdomains. The catalytic subdomain (palm) coordinates two metal-ions and mediates the nucleotidyltransferase reaction [114] and the other subdomains mediate the binding of duplex DNA (thumb) and nascent base pairs (fingers) [115]. It has been shown that DNA Pol β is able to fill short gaps in double stranded DNA [116]. The 8 kDa lyase domain was shown to direct DNA Pol β to phosphorylated 5' side of a DNA gap for its bypass [117] and recently to mediate the removal of 5' dRP from the AP site via β-elimination after the incision step by the AP endonuclease [118]. Beside the role of DNA Pol β in short patch BER, a function in long patch BER was proposed. Both short- and long-patch repair are impaired after DNA Pol β ablation [119-121]. Additionally, DNA Pol β has a role in bypassing DNA lesions such as cisplatin-DNA adducts [122]. It was shown that DNA Pol β is not involved in the diversification of IgG [123]. DNA Pol β knockout mice are not viable reflecting the important role of Pol β during embryonic development [124].

Down-regulation of DNA Pol β sensitized mouse fibroblasts to cisplatin, UV-irradiation, oxidizing- and methylating agents [125-128]. Ectopic expression of DNA Pol β leads to aneuploidy, aberrant localization of the centrosome-localized γ-tubulin protein during mitosis, checkpoints defects *in vitro *and tumour induction *in vivo *[129]. In addition, DNA Pol β expression is upregulated in chronic myelogenous leukemia (CML) patients [130]. Thus, a strict regulation of DNA Pol β activity is essential to maintain genomic integrity.

### TLS Pol λ

TLS Pol λ has a size of approximately 69 kDa and its gene *POLL *is located on the chromosome 10 in human and on the chromosome 19 in mice [33,35]. The human TLS Pol λ consists of 575 aa and shares 32% residue identity with Pol β, comprising a C-terminal Pol domain including palm, thumb and fingers and the 8 kDa 5' dRP lyase domain. Additionally, TLS Pol λ contains an N-terminal BRCT (BRCA1 C-terminus) domain followed by a serine/proline rich region that is absent in Pol β [131]. Tandem BRCT domains mediate binding to phosphorylated proteins and are widely found in proteins involved in DDR [132]. TLS Pol λ shows terminal TdT activity [131], which prefers the incorporation of pyrimidine nucleotides [133]. The TLS Pol λ has been shown to be less accurate for base substitutions and much less accurate for single-base deletions [134]. Further, TLS Pol λ is unable to differentiate between matched and mismatched primer termini during the extension step, therefore suggesting TLS Pol λ as a candidate for NHEJ and as mismatch extender during TLS [134,135]. Additional *in vitro *studies showed that TLS by Pol λ requires the BRCT domain and is physically and functionally dependent on Ku during NHEJ [136]. Recently, it has been shown, that a TLS Pol λ variant containing of a single nucleotide polymorphism (SNP), a cytosine/thymine variation, leads to increased mutation frequency, chromosomal aberration and defects in NHEJ [137].

TLS Pol λ is also discussed to participate in BER. Uracil-containing DNA was efficiently repaired in an *in vitro *reconstituted BER reaction by the 5' dRP lyase activity of TLS Pol λ, in coordination with its polymerization activity [138]. TLS Pol λ null mice are viable and fertile, but shortening of the heavy chain coding joints was reported [49].

### TLS Pol μ

TLS Pol μ has a size of 55 kDa and its gene *POLM *is located on the human chromosome 7. It consists of 492 aa and shares 42% identity with TdT [33,35]. TLS Pol μ, as TLS Pol λ, contains a polymerase domain and a BRTC domain. In contrast to Pol β and λ, the TLS Pol μ lacks a 5' dRP lyase activity [138]. Isolated TLS Pol μ is highly error-prone for frameshifts during DNA synthesis. Interestingly, TLS Pol μ is able to extend from mismatches by frameshift synthesis mechanism and thereby promoting microhomology search and microhomology pairing between the primer and the DNA template [139]. Moreover, TLS Pol μ shows template-independent polymerase activity under physiological conditions (Mg^2+ ^present) preferring the incorporation of pyrimidines and thereby generating terminal microhomology, which can be ligated by the XRCC4-DNA ligase IV [140]. All these findings suggest TLS Pol μ as a candidate for NHEJ of DSBs. It has been shown that TLS Pol μ, as TLS Pol λ, interacts with the Ku-DNA complex through its BRCT domain [136].

Interestingly, TLS Pol μ null mice are viable and fertile, but they show impaired V(D)J recombination due to shortening of the light chain coding ends, but not of the heavy chain coding ends [49,50].

### Family Y: Rev1, TLS Pol eta (η), kappa (k) and iota (ι)

The human TLS Pol members of the Y family include REV1, TLS Pol η, TLS Pol k and TLS Pol ι [65]. All the Y family members lack the 3' to 5' exonuclease proofreading activity [56] and share a general conserved N-terminal polymerase domain for the catalytic activity and a non-conserved C-terminus, which, at least for the human TLS Pol ι [141] and Pol κ [142], is responsible for the regulation of the activity. The conserved N-terminus of the DNA polymerase domain includes five motifs (I to V) corresponding to the catalytic core complex. Motif I and II form the catalytic epicentre (palm) with its three acidic residues harbouring the two metal ions mediating the nucleotide transfer. Despite sequence differences, the palm domain with its nucleotide transfer function is widely conserved between Y-family TLS, A- and B-family replicative DNA polymerases. The motifs III and IV belong to the finger and thumb domain, respectively. They bind the triphosphate of the nascent incoming dNTP and mediate the incorporation of the nascent nucleotide whereas the additional motif V binds the primer strand. The C-terminus of the motif V resides either the so called little finger (LF), polymerase associated domain (PAD) or the wrist that supports the DNA synthesis activity and is conserved and unique among the Y-family TLS polymerases [53,143,144].

### Rev1

The 1251 aa human REV1 protein has a size of 138 kDa and is encoded by the gene REV1, located on chromosome 2. Beneath the typical Y-family conserved domains, a BRCT domain is located at the N-terminus [132]. At the C-terminal end, there are two ubiquitin binding motifs (UBM) [145] followed by a polymerase interaction region [146].

The polymerase activity of Rev1 is restricted to the incorporation of C over G and DNA lesions such as AP sites [147]. It has been proposed that the nucleotide insertion activity is not the main function of REV1 but that REV1 helps to coordinate the polymerase switch between the normal- and the substituting TLS Pol upon PCNA monoubiquitination. Murine Rev1 binds ubiquitin through its UBMs and thereby mediating its localization to DNA damage foci. UBM mutants showed increased mutational aberrations after UV irradiation and elevated sensitivity to UV irradiation and cisplatin, which was further increased in UBM and BRCT double-mutants [148]. Although it was shown that murine REV1 binds monoubiquitinated PCNA via its UBMs, it is assumed that the polymerase switch function of REV1 might also be dependent on the other protein interaction domains of REV1, e.g. the BRCT and the polymerase interaction region. In detail, the C-terminal end of human Rev1 is able to interact with several TLS polymerases including Pol η, Pol ι, Pol κ and Pol ζ, thus supporting the assumption that Rev1 acts as a scaffold protein for several TLS polymerases [107,146,149]. Additionally, murine REV1 binds PCNA through its BRCT domain and the monoubiquitination of PCNA enhances this reaction [150].

Rev1 ablation sensitizes DT40 chicken cells to various DNA damaging agents including cisplatin, UV irradiation and MMS. Additionally, Rev1 is required for the maintenance of chromosomal stability after UV irradiation [150]. Rev1 deletion in chicken DT40 cells did not affect basal and damage induced sister chromatid exchange and immunoglobulin gene conversion indicating that homologous recombination repair is likely to be intact [151]. However, the same study showed that knockout of Rev1 in chicken DT40 cells reduced the level of non-templated immunoglobulin gene mutations indicating a defect in translesion bypass of DNA replication blocking lesions. An *in vivo *mouse model showed that REV1 inhibition in B-cell lymphoma reduces cisplatin and cyclophosphamide induced mutagenesis and prolongs survival of mice upon cyclophosphamide treatment [102].

Recently, it has been shown that Rev1 silencing impairs the replication of G-quadruplex (G4) structures thereby, on one side, limiting the recycling of histones and, on the other side, favouring the incorporation of newly synthesized histones resulting in changes of the epigenetic pattern, e.g. gene silencing [152].

### TLS Pol η

Loss of TLS Pol η activity in human results in a cancer-prone syndrome known as xeroderma pigmentosum variant (XPV), which is characterized by an increased incidence of skin cancers and sensitivity to sunlight [153]. Human TLS Pol η consists of 713 aa and is encoded by the *POLH *(Xeroderma pigmentosum variant, XPV) gene, localized on chromosome 6. TLS Pol η has a size of 78 kDa. Additional to the N-terminal conserved polymerase domain, TLS Pol η consists of a Rev1-interacting region (RIR), an ubiquitin binding zinc finger (UBZ), a nuclear localization domain (NLD) and two PIPs [154]. XPV cells are sensitive to UV irradiation and show an increased mutagenic rate despite functional NER indicating that TLS Pol η bypasses specific UV lesions in a non-mutagenic manner [41,57,155]. It is proposed that in the absence of TLS Pol η, TLS Pol ι serves as the error-prone polymerase which bypasses the UV-induced lesions [156]. Also Rev1 is suggested to have a regulatory role in TLS of UV-induced lesions [157]. The PIP and the UBZ domain of TLS Pol η are essential for binding the monoubiquitinated PCNA and its TLS activity. Mutation in either the PIP or the UBZ domain increases the UV sensitivity [145].

Interestingly, it has been shown that loss of TLS Pol η in mice leads to decrease in adenine/thymine mutations during SHM of IgG indicating that TLS Pol η bypasses adenine and thymine in an error prone manner [158].

### TLS Pol κ

The human TLS Pol κ is encoded by *POLK *gene and has a length of 870 aa. The TLS Pol κ has a size of 99 kDa and is located on chromosome 5. The N-terminal part consists of the conserved polymerase domain whereas the variable C-terminus consists of a RIR, two UBZ and a PIP. It has been shown that TLS Pol κ co-localizes to a lesser extend with PCNA at replication foci after UV irradiation, hydroxyurea or BaP treatment compared to TLS Pol η [159]. It has been shown that embryonic stem (ES) cells deficient in the TLS Pol κ gene are more sensitive and acquire more mutations after treatment with BaP and that TLS Pol κ bypasses BaP-G accurately and efficiently *in vivo *[45]. Additionally, XPV cells treated with siRNA against TLS Pol κ reveal increased UV sensitivity [160] indicating that TLS Pol κ is able to bypass UV-induced DNA lesions. Recent findings suggest that TLS Pol κ has a function during NER and that its activity is dependent on RAD18 and monoubiquitinated PCNA [161].

A role of TLS Pol κ in promoting tumorigenesis has been discussed since ectopic expression of Pol κ leads to DSBs, aneuploidy and tumorigenesis in nude mice [162].

### TLS Pol ι

Human TLS Pol ι is encoded by the *POLI *gene consists of 715 aa. The TLS Pol ι has a size of 80 kDa and is localized on chromosome 18. TLS Pol ι shares with the other Y family members the N terminal conserved polymerase domain and at the variable C-terminal a RIR, two UBMs and the PIP. Interestingly, TLS Pol ι possesses a 5' dRP lyase activity [163] that is located within a NLD [164].

As in the case of TLS Pol η, the PIP and the UBM domains of TLS Pol ι are important for localization to the replication fork by binding monoubiquitinated PCNA [145]. The localization and accumulation of TLS Pol ι to stalled replication forks is dependent on physical interaction with TLS Pol η [165]. Recently, it has been shown that BER activity is decreased in human fibroblasts in which TLS Pol ι is stably downregulated resulting in increased sensitivity to the oxidizing agents H_2_O_2 _and menadione [164]. Additionally, after H_2_O_2 _treatment, TLS Pol ι binds to chromatin and interacts with the BER factor XRCC1, suggesting a role of TLS Pol ι not only in TLS but also in the repair process of oxidative DNA damage [164].

### Activation of TLS and polymerase switch reaction

In response to DNA damage, it is proposed that activation of the DNA damage tolerance mechanisms is mainly mediated by modifications of PCNA [166,167]. Monoubiquitination of PCNA by the RAD6-RAD18 complex triggers DNA damage tolerance by TLS. Y-family TLS polymerases can bind to monoubiquitinated PCNA through ubiquitin-binding domains such as the UBM, the UBZ and a PCNA interacting peptide box (PIP), thereby initiating TLS [145,148].

In *S. cerevisiae*, monoubiquitinated PCNA is subsequently polyubiquitinated by RAD5/Ubc13/Mms2, which triggers the error-free DNA damage tolerance response carried out by template switching including fork reversal or recombination past the lesion [168,169]. There are two Rad5 orthologs in humans, HLTF and SHPRH, which are both capable of polyubiquitinating PCNA *in vitro *[170,171]. An interplaying mechanism was proposed between the deubiquitinating enzyme USP1 and ubiquitination factors including RAD6 and RAD18. USP1 acts as a negative regulator, thus removing the ubiquitin residue from the monoubiquitinated PCNA to reduce the mutagenic effect of TLS polymerases [172,173].

Studies in yeast indicate that other factors than PCNA might be involved in the recruitment of TLS polymerases to damaged sites and the subsequent polymerase exchange reaction. Similar to PCNA, the Fanconia anaemia (FA)-ID complex might also promote the exchange of replicative DNA polymerases by TLS polymerases after replication fork blockage [174-176]. The yeast Rad9-Rad1-Hus1 (9-1-1) checkpoint clamp, which is loaded by the RAD24-replication factor C (RFC) clamp loader, physically interacts with Pol ζ indicating a putative role of the (9-1-1) checkpoint clamp in the recruitment of TLS polymerases after checkpoint activation [177].

It was proposed that the major function of TLS polymerases is to allow replication to continue in the presence of DNA damage during S-phase [178]. However, subsequent studies in yeast revealed that TLS polymerases also have a function in post-replicative gap filling, the so called post replicative repair (PRR) [179]. PRR is not necessarily temporally separated from S-phase. Yeast REV1, which interacts and thereby regulates the activity of several TLS polymerases, is highly expressed in late S and early G_2 _phase [180]. Additionally, studies in mammalian U2OS cells revealed that human REV3 accumulates in G_1_-phase and at the G_2_/M transition [97]. In addition, it was show that replication-dependent TLS across UV-adducts is not affected in REV3-deficient MEFs. However, although a significant fraction of CPDs were still bypassed, post-replicative repair of [6-4]PPs was completely abolished in REV3-deficient MEFs [181]. Recently, it was shown in chicken DT40 cells that REV1 is essential to maintain normal replication fork progression in the presence of replication-blocking DNA lesions whereas PCNA is required for post-replicative gap filling [182].

Taken together, the activation of the DNA damage tolerance pathway is regulated by the mono- or polyubiquitination of PCNA, REV1, and the FA-ID complex, and might also dependent on the stage of the cell cycle. The regulation of TLS and post-replicative repair recently got into the limelight but more studies are needed to fully elucidate how TLS is regulated during DDR.

### One- and two-polymerase mechanism

**Figure 3:**
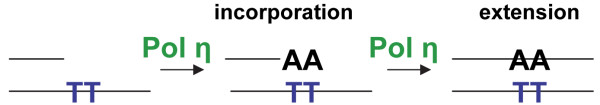
**One- polymerase error-free bypass of a UV-induced TT-CPD carried out by TLS Pol η (adapted from **[247]). See text for details.

Some TLS polymerases are able to autonomously replicate over a DNA lesion by both incorporating nucleotides opposite the damaged DNA and by extending from the inserted nucleotides. This so called one- polymerase mechanism has been shown to be performed by TLS Pol κ replicating over AP sites *in vitro *and by TLS Pol η bypassing UV-induced CPDs *in vivo*. Interestingly, CPDs, the main DNA lesions induced by both UVB and UVA radiation, are bypassed by Pol η in an error-free manner (Figure 3).

**Figure 4:**
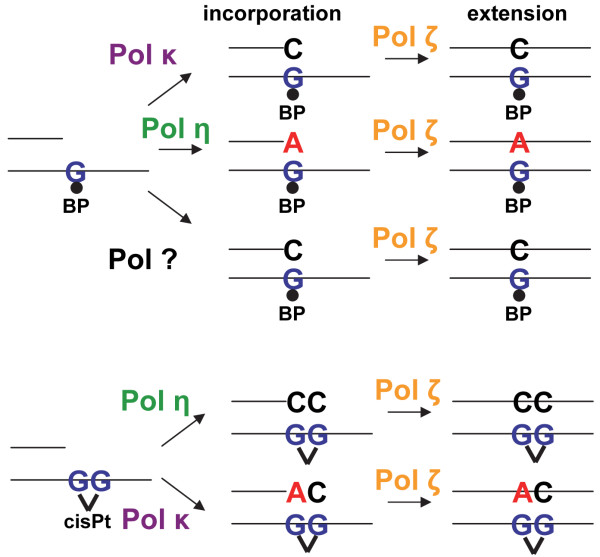
**Two-polymerase mechanisms for bypassing BaP-G (top) or cisplatin (cisPt) (bottom) DNA adducts**. The insertion step is performed by one or a combination of several TLS polymerases incorporating nucleotides in an error-free or error-prone manner opposite the adduct whereas the extension step is mainly carried out by TLS Pol ζ (adapted from [247]). See text for details.

However, other lesions such as BaP-G or cisplatin-DNA adducts are mainly bypassed in a process requiring the continuous processing by two TLS polymerases, a so called two-polymerase mechanism. The first TLS polymerase incorporates the nucleotides opposite the DNA lesion and a second TLS polymerase subsequently extends from the inserted nucleotide. Depending on the type of DNA lesion, different pairs of TLS polymerases interact together to replicate the DNA lesion resulting in either an error-free or error-prone bypass (Figure 4).

### DNA damage specific TLS

A variety of different DNA damages can result in the arrest of DNA replication, subsequently requiring TLS to avoid the conversion of a stalled replication fork or post-replication gap into a genotoxic DNA double strand break. Thus, TLS is essential to maintain the genomic integrity and it is therefore not surprising that the system is redundant, i.e. every kind of DNA damage can be bypassed by various combinations of one- or two TLS polymerases (Table 1).

### DNA apurinic/apyrimidinic (AP) sites

Nonenzymatic hydrolysis of the base-sugar bonds in DNA and the accumulation of BER-intermediates results in the formation of an estimated 10'000 AP sites per human cell per day [4]. Ionizing radiation and bleomycin, which both are used to treat various types of cancers, not only induce cytotoxic DNA DSBs but also AP sites [183]. AP sites are processed by BER and Pol β is the primary enzyme used for gap filling DNA synthesis during BER. If not repaired, AP sites can be bypassed by TLS polymerases. However, TLS across an AP site is highly error-prone since the sequence information of an AP site is missing. It was shown that TLS Pol β can bypass an AP site resulting in deletions and base substituting errors [184,185]. A one-polymerase mechanism has been proposed based on an *in vitro *assay where the TLS Pol θ preferentially incorporates an adenine opposite the AP site followed by a guanine (G) and cytosine/thymine (C/T) [62]. An A opposite the AP site is also the best primer for the extension step by TLS Pol θ [62]. In addition, it has been shown *in vitro *that TLS Pol η is able to incorporate nucleotides opposite AP sites preferentially A and G and extend from the incorporated nucleotide favouring an A [186]. *In vitro *studies showed that isolated TLS Pol λ from calf thymus is able to replicate a DNA template containing an AP site *in vitro *[187]. Similarly, isolated human TLS Pol λ is able to synthesize over an AP site and this bypass is stimulated by PCNA *in vitro *[188]. TLS Pol κ is able to autonomously bypass AP sites *in vitro *[189]. Similarly, TLS Pol μ is capable to incorporate nucleotides opposite AP sites *in vitro *although deletions are frequently generated due to primer realignment [190]. Additionally, The replicative DNA Pol δ in the presence of PCNA preferentially inserted A across AP sites and is also able to extend from the lesion [191].

Alternatively, AP sites might also be bypassed by a two-polymerase mechanism. TLS Pol ι and REV1 are able to incorporate one nucleotide opposite an AP site but the extending polymerase was not identified [191].

### 7, 8-dihydro-8-oxoguanine (8-oxo-G)

Oxidative stress can lead to the generation of reactive oxygen species (ROS), which induce base modifications such as 8-oxo-G and thymine glycol [192,193]. Studies have shown an increased level of oxidative DNA damage in cancerous tissue, e.g. a 9-fold increase of 8-oxo-G in tissue from breast cancer compared to surrounding normal tissue [194]. 8-oxo-G is generated by oxidative stress and leads to frequent misincorporation (10-75%) of adenine by human replicative DNA polymerases generating a G:C to T:A transversion [195]. In addition, TLS Pol κ also inserts mainly adenine opposite 8-oxo-G [196]. *In vitro *experiments revealed that TLS Pol ι is able to bypass 8-oxo-G with low efficiency in a generally non-mutagenic manner thereby preferring the incorporation of C over 8-oxo-G followed by A and G [197]. It has been shown that 8-oxo-G lesion *in vitro *can be bypassed by TLS Pol μ resulting in a -1 deletion due to primer realignment during TLS [190]. More recently, it has been shown *in vitro *that 8-oxo-G lesions are mainly bypassed by TLS Pol λ and TLS Pol η and that the presence of PCNA and RP-A increases the fidelity of correct C incorporation over the incorrect A incorporation opposite the 8-oxo-G 1200-fold for TLS Pol λ and 68-fold for TLS Pol η [198]. Additionally, PCNA and RP-A inhibited error-prone TLS opposite an 8-oxoG by DNA polyerase β [199].

### Thymine glycol

Thymine glycol is the most common thymine lesion induced by reactive oxygen species (ROS). *In vitro *studies have shown, that TLS Pol θ is able to incorporate nucleotides opposite both 5R- and 5S-diastereoisomers of thymine glycol with similar efficiency but fails to process the extension step [62]. TLS Pol ν is able to bypass 5S-thymine glycol in an error-free manner whereas the bypass of 5R-thymine glycol was less accurate [51]. TLS Pol β and TLS Pol λ have been shown to bypass thymine glycols in gapped DNA structures. Additionally, dependent on the size of the gap, TLS Pol λ is able to perform the extension step. The bypass fidelity of TLS Pol λ is increased by the presence of PCNA [200]. Recently, a two-polymerase mechanism for error-free thymine glycol bypass including TLS Pol κ as nucleotide inserter and TLS Pol ζ as extender was proposed [201].

### [6-4]pyrimidine-pyrimidone photoproduct ([6-4]PP)

The most abundant environmental source of DNA damage is UV-light, which induces nucleotide dimerization, e.g. CPDs and [6-4]PPs at a 3:1 ratio [202]. One hour at the beach results in the induction of approximately 1 × 10^5 ^UV-adducts per exposed cell [203].

The induction of [6-4]PPs induces a bend of 44° in the DNA helix, which triggers the efficient recognition and repair of [6-4]PPs by NER [204]. In a primer extension assay, only TLS Pol η was able to autonomously insert nucleotides opposite an [6-4]PP although with a significant lower efficiency than opposite CPDs and without detectable extension [51]. Replication-dependent bypass of UV-adducts was not delayed in REV3-deficient MEFs. However, post-replicative repair of [6-4]PPs was completely dependent on REV3, i.e. TLS Pol ζ [181]. Based on a *in vivo *plasmid assay, alternative two-polymerase mechanism models have been proposed for the bypass of [6-4]PPs. In the first model, TLS Pol ι and TLS Pol η alternatively incorporate nucleotides opposite a [6-4]PP in a process, which is error-free or error-prone at the 3' thymine and 3' cytosine, respectively, and which results in the subsequent extension by a yet unknown DNA TLS polymerase. In the second model, a yet unknown polymerase incorporates nucleotides opposite a [6-4]PP in a process, which is error-free at the 3' thymine or 3'cytosine. Subsequent extension is carried out by TLS Pol ζ [205]. An additional two-step model was proposed where TLS Pol ι incorporates nucleotides opposite a [6-4]PP in an error-prone manner and TLS Pol θ carries out the subsequent extension step from the mismatched primer terminus [70].

### Cyclobutane pyrimidine dimer (CPD)

CPDs are not as much DNA helix distorting (9°) as [6-4]PPs and are therefore not an ideal substrate for NER [206]. Hence, CPDs persist longer after UV-irradiation than [6-4]PPs thereby blocking DNA replication more frequently thus rendering their tolerance more dependent on functional TLS. Depletion of TLS Pol η renders human cells sensitive to UV-irradiation, especially to CPD induction [41,160]. TLS Pol η is able to replicate error-free over CPDs *in vitro *[207], with a higher error rate at the 3'T than at the 5'T [208].It has been shown *in vivo *that TLS was reduced and mutagenicity increased in cells lacking TLS Pol η using a quantitative TLS assay measuring TLS across CPDs. Also *in vivo*, most of the mutations were found opposite the 3'T of the CPD [209].

Subsequently, it has been proposed that CPDs in XPV cells, e.g. in the absence of TLS Pol η, are replicated by the two-polymerase mechanism in an error-prone manner. The two step model includes either TLS Pol ι, TLS Pol κ or a yet undefined polymerase or their combined action for the first and the second pyrimidine nucleotide incorporation opposite a CPD, followed by the extension step achieved by TLS Pol ζ and to a minor extend by TLS Pol κ [160]. Additionally, i*n vitro *experiments revealed that TLS Pol μ can autonomously bypass CPDs in a mainly error-free manner and that the subsequent extension was further enhanced by TLS Pol ζ [190].

### Benzo[α]pyrene-guanine (BaP-G)

BaP is a major compound of tobacco smoke and forms, upon metabolic activation, a covalent BaP-G DNA adduct, which is associated with the development of lung cancer [210]. BaP-G frequently mispairs during DNA replication with A therefore leading to G:T transversions [210]. *In vitro *and *in vivo *studies showed efficient bypass of BaP-G by TLS Pol κ using a gapped plasmid containing a BaP-G lesion [211]. Additionally, BaP-G has been shown to be bypassed by a two-polymerase mechanism *in vivo*. TLS Pol κ inserts nucleotides opposite a BaP-G error-free whereas insertion by TLS Pol η is error-prone. Subsequent extension is performed by TLS Pol ζ. Since the combined inhibition of TLS Pol κ and TLS Pol η did not decrease TLS to a similar extend than depletion of TLS Pol ζ, it was suggested that a third unknown TLS polymerase might be involved in insertion opposite a BaP-G DNA adduct [64]. Indeed, it was shown that TLS Pol μ was able to bypass bulky DNA lesions including BaP-G DNA adducts [190].

### Intrastrand-crosslinks

The chemical agent cisplatin is used for therapeutical treatment of most solid tumors including lung cancer and malignant pleural mesothelioma and forms DNA intra- and interstrand-crosslinks (ICLs), which can lead to a blockage of the DNA replication machinery [6]. Intrastrand-crosslinks are the most prevalent form of cisplatin-induced DNA adducts (> 90%) [6] and are bypassed by one- or the two-polymerase mechanisms. *In vitro *experiments revealed that Pol β can bypass various cisplatin intrastrand adducts [122,212]. It has been reported that TLS Pol η *in vitro *is able to bypass a d(GpG)-cisplatin intrastrand adduct thereby preferentially incorporating C opposite the d(GpG)-cisplatin intrastrand adduct [186,213]. TLS Pol ζ can also bypass cisplatin intrastrand adducts although with low efficiency [214]. It was subsequently shown *in vitro *that TLS Pol μ is less efficient than pol η in catalyzing translesion synthesis past platinum intrastrand adducts but appears to be significantly more efficient than Pol β or Pol ζ [215]. Recently, it has been shown *in vivo*, that either TLS Pol η or TLS Pol κ incorporate the correct or incorrect, respectively, nucleotide opposite the d(GpG)-cisplatin intrastrand adduct and TLS Pol ζ carries out the extension step [64]. Similarly, *in vivo *experiments proposed a model where RAD18/RAD6 dependent monoubiquitination of PCNA activates the bypass of the d(GpG)-cisplatin intrastrand adduct by TLS Pol η and activated TLS Pol ζ performs the subsequent extension in a REV1-dependent manner [105].

### Interstrand-crosslink (ICL)

An excellent review of ICL repair and cancer has been published recently [216]. In contrast to the DNA damages described above, ICLs cannot be bypassed since, as the name implies, both DNA strands are covalently linked and therefore no template for DNA synthesis is available. It was proposed that ICLs kill cells due to 1.) Blockage of DNA replication 2.) Stalling of transcription or 3.) Distortion of the chromatin thereby preventing the access of DNA-interacting proteins (reviewed in [216]). Thus, a complex ICL repair machinery evolved and it was shown that TLS polymerases are involved in this process.

Lipid peroxidation can occur inside the body or in foods before they are eaten resulting in the production of by-products capable of crosslinking DNA, e.g. β-unsaturated aldehydes [217]. Recent biochemical and cellular studies implicated that dialdehydes formed by lipid peroxidation induce minor groove ICLs, which are repaired by a TLS Pol κ dependent mechanism [218]. In cancer therapy, cisplatin is the most widely used crosslinking drug but only approximately 10% of the total DNA adducts induced by cisplatin are ICLs [6]. XPV cells, which are deficient for TLS Pol η, are hypersensitive to cisplatin [219,220].

During the G_1 _phase of the cell cycle, ICL are repaired in a recombination-independent pathway including NER, TLS Pol ζ and Rev1 [221]. Experiments with *X. laevis *egg extracts revealed that during replication-dependent ICL repair, the replicative DNA polymerase stalls about 24 nucleotides before the crosslink [222]. At this site, the replicative DNA polymerase is replaced by a TLS polymerase, most probably TLS Pol ν [76], which extends the nascent strand to within 1 base pair of the ICL. Subsequently, DNA nucleases unhook the ICL and REV1 inserts a cytosine opposite the unhooked ICL [223,224]. Several of the TLS polymerases may extend DNA synthesis beyond the ICL but only deletion of TLS Pol ζ abrogates TLS extension in *X. laevis *extracts and is was suggested that only Pol ν, REV1 and Pol ζ are essential for ICL repair (reviewed in [216]). Indeed, a recent study showed in human cells that only REV1 and TLS Pol ζ are required for the repair of ICLs whereas RAD18, TLS Pol η, REV1 and TLS Pol ζ are all necessary for replicative bypass of cisplatin intrastrand DNA crosslinks [175].

### Relevance of TLS polymerases in cancer therapy

**Figure 5:**
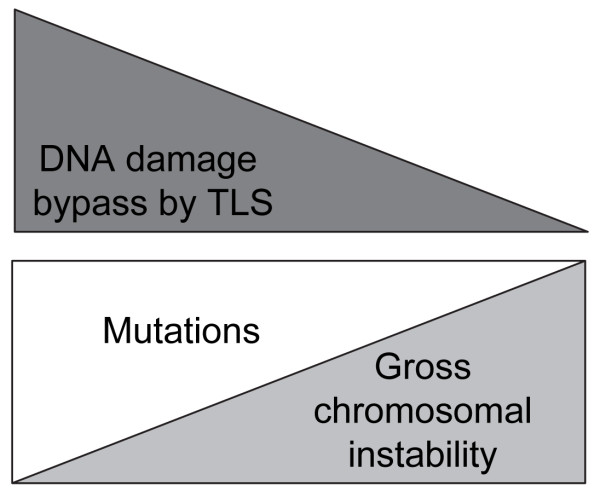
**Trade-off between the accumulation of mutations due to DNA lesion bypass by TLS and the accumulation of genomic instability in the absence of TLS**.

Targeting the error-prone Pol ζ by deletion of *REV3 *in yeast results in a reduced spontaneous mutation rate [225]. On the other hand, selective deletion of *REV3 *in mature B cells impaired proliferation and genomic stability [106]. Thus, as indicated in Figure 5, a reduction of the mutation rate by inhibition of TLS is inversely correlated with an increase in gross chromosomal instability. Since genomic instability is a hallmark of cancer, an important question is how carcinogenesis is influenced by changes in the expression or activity of TLS polymerases. XPV patients who are deficient for TLS Pol η activity, suffer from a very high cancer incidence since TT-CPDs, which are bypassed error-free by Pol η, are bypassed in the absence of Pol η by alternative TLS polymerases in an error-prone manner [160]. In addition, somatic mutations of Pol β were identified in adenocarcinoma of the colon [226] and mutations in the gene enconding Pol ι are associated with increased susceptibility to lung cancer in mice [10,227,228] and humans [229]. Single nucleotide polymorphisms in the human *REV1 *gene are associated with increased lung cancer risk [229]. Changes in expression and mutations in the genes encoding Pol ι and Pol κ have been found in human tumors [230-232]. Gene expression of TLS Pol θ is upregulated in two cohorts of patients with untreated primary breast cancers, which correlates with poor clinical outcome [233]. In summary, there is a growing body of literature indicating that increased as well as decreased TLS activity is associated with increased and/or accelerated tumorigenesis.

However, transient inhibition of TLS activity might be beneficial for cancer treatment. In detail, the concept of "synthetic lethality" is applied as a therapeutic approach where defects in two pathways alone can be tolerated but become lethal when combined. DDR is often abrogated in cancer cells and it was proposed to develop cancer treatments taking advantage of cancer-specific DDR alterations [234]. The principle of synthetic lethality was successfully applied in cancer therapy of patients carrying mutations in *BRCA1 *or *BRCA2*, a specific DNA-repair defect. Inhibition of poly(adenosine diphosphate [ADP]-ribose) polymerase (PARP) resulted in synergistic antitumor activity in the treatment of hereditary ovarian- and breast cancer of patients with *BRCA *mutations [235].

In analogy, there are indications that targeting TLS polymerases *per se*, i.e. without additional chemotherapy, might be applicable for cancer therapy. For example, based on the overlap in function of mismatch repair and DNA polymerase proofreading activity, it was recently shown that inhibition of Pol β or γ induces synthetic sickness/lethality in MSH2- or MLH1-deficient human cancer cells, respectively [236]. Similarly, a recent study from our laboratory revealed that inhibition of *REV3 *expression *per se *resulted in decreased colony formation and accumulation of persistent DNA damage in cancer cell lines of different origin whereas cell growth of control cell lines was less affected [237].

In addition, it was proposed that targeting TLS should enhance the therapeutic effect and reduce the resistance formation of DNA-damaging chemotherapeutics [238]. Indeed, inhibition of *REV3 *expression sensitized human fibroblasts to cisplatin and decreased the formation of cisplatin resistant cells *in vitro *[95]. Recent findings in a transplantable mouse xenograft model showed that suppression of *REV3 *expression increased the sensitivity of chemoresistant adenocarcinomas to cisplatin and reduced occurrence of cisplatin-induced resistance [103]. Similarly, using a preclinical mouse model of Burkitt's lymphoma, it was shown that suppression of both *REV1 *and *REV3 *expression, sensitized lymphomas to cisplatin [102]. The same study also showed that *REV1 *suppression in lymphoma cells inhibited resistance formation after cyclophosphamide treatment *in vitro *and improved cyclophosphamide-based chemotherapy of lymphomas *in vivo*.

Interestingly, studies in yeast showed that resistance formation after hydroxyurea treatment, which inhibits the ribonucleotide reductase and therefore reduces/imbalances the nucleotide pool, is also suppressed by *REV3 *deletion [239]. Thus, suppression of TLS polymerases might also reduce resistance formation of drugs whose therapeutic effect is also based on the reduction/imbalance of the nucleotide pool, e.g. gemcitabine and pemetrexed. In summary, inhibition of the expression/activity of TLS activity may increase responsiveness to genotoxic treatments and improve the clinical outcome.

Although the effects of TLS inhibition on cancer cells are well investigated, less is known how inhibition of TLS affects normal cells. No deficiency in cell growth/survival was mentioned after antisense-based inhibition of *REV3 *expression in human non-tumor cell lines [66,100,237]. In contrast, it was shown by different groups that *REV3 *knockout reduced cell growth of MEFs [92,93]. Thus, it will be crucial to carefully evaluate how normal cells are affected by any cancer therapy based on TLS inhibition. To minimize the risk of malignant transformation of normal cells due to the induction of genomic instability by TLS inhibition during cancer therapy, transient inhibition would be favourable over long-term inhibition, e.g. specific small molecules inhibitors would be favourable over a lentiviral-based system constitutively delivering a TLS polymerase-targeting siRNA.

To date, no specific inhibitors for Y family TLS polymerases are available except for the pyrene nucleotide analogs oxetanocin (OXT)-GTP and -ATP, which are able to inhibit TLS Pol η [240]. Additionally, some natural inhibitors are known to have inhibitory effects such as Petasiphenol, which is a specific inhibitor of TLS Pol λ *in vitro *[241]. It has been shown that Petasiphenol has antiangiogenic activity [242,243]. Tormetic acid is another inhibitor of TLS Pol λ and β but also of replicative DNA polymerases, e.g. Pol α. Tormetic acid showed an antitumorigenic activity *in vivo *[243].

## Conclusions

The existence of error-prone TLS polymerases reflects a trade-off between avoiding gross chromosomal instability due to replication fork breakdown and the occurrence of mutations on the nucleotide level (Figure 5).

It was proposed that the evolution of long lived and large animals such as vertebrates necessarily entailed the acquisition of potent tumor suppressive mechanisms [244]. Thus, compared to lower organisms such as bacteria and yeast, it can be speculated that the increased number of TLS polymerases evolved in higher organisms as a tumor suppressive adaptation. Alternatively, research carried out in the last years showed that mammalian TLS polymerases are not only involved in bypassing and repair of DNA lesions but also in the diversification processes of IgG and the maintenance of epigenetic modifications. In summary, TLS polymerases have a function beyond the maintenance of the genomic integrity and it will be interesting tofurther elucidate the involvement of TLS in the delicate balance between cancer suppression and longevity.

## Competing interests

The authors declare that they have no competing interests.

## Authors' contributions

PAK and TMM were both involved in writing and editing the manuscript. Both authors read and approved the final manuscript.

## References

[B1] Lander ES Linton LM Birren B Nusbaum C Zody MC Baldwin J Devon K Dewar K Doyle M FitzHugh W Initial sequencing and analysis of the human genome Nature 2001 409 6822 860 921 10.1038/35057062 11237011

[B2] Venter JC Adams MD Myers EW Li PW Mural RJ Sutton GG Smith HO Yandell M Evans CA Holt RA The sequence of the human genome Science 2001 291 5507 1304 1351 10.1126/science.1058040 11181995

[B3] Lindahl T Barnes DE Repair of endogenous DNA damage Cold Spring Harb Symp Quant Biol 2000 65 127 133 10.1101/sqb.2000.65.127 12760027

[B4] Barnes DE Lindahl T Repair and genetic consequences of endogenous DNA base damage in mammalian cells Annu Rev Genet 2004 38 445 476 10.1146/annurev.genet.38.072902.092448 15568983

[B5] Hecht SS Tobacco smoke carcinogens and lung cancer J Natl Cancer Inst 1999 91 14 1194 1210 10.1093/jnci/91.14.1194 10413421

[B6] Zamble DB Lippard SJ Cisplatin and DNA repair in cancer chemotherapy Trends Biochem Sci 1995 20 10 435 439 10.1016/s0968-0004(00)89095-7 8533159

[B7] Aguilera A Gomez-Gonzalez B Genome instability: a mechanistic view of its causes and consequences Nat Rev Genet 2008 9 3 204 217 10.1038/nrg2268 18227811

[B8] Chuang PT Kawcak T McMahon AP Feedback control of mammalian Hedgehog signaling by the Hedgehog-binding protein, Hip1, modulates Fgf signaling during branching morphogenesis of the lung Genes Dev 2003 17 3 342 347 10.1101/gad.1026303 PMC195990 12569124

[B9] van der Wilt CL Backus HH Smid K Comijn L Veerman G Wouters D Voorn DA Priest DG Bunni MA Mitchell F Modulation of both endogenous folates and thymidine enhance the therapeutic efficacy of thymidylate synthase inhibitors Cancer Res 2001 61 9 3675 3681 11325838

[B10] Lee GH Nishimori H Sasaki Y Matsushita H Kitagawa T Tokino T Analysis of lung tumorigenesis in chimeric mice indicates the Pulmonary adenoma resistance 2 (Par2) locus to operate in the tumor-initiation stage in a cell-autonomous manner: detection of polymorphisms in the Poli gene as a candidate for Par2 Oncogene 2003 22 15 2374 2382 10.1038/sj.onc.1206387 12700672

[B11] Broomfield S Hryciw T Xiao W DNA postreplication repair and mutagenesis in Saccharomyces cerevisiae Mutation research 2001 486 3 167 184 10.1016/s0921-8777(01)00091-x 11459630

[B12] Bessman MJ Kornberg A Lehman IR Simms ES Enzymic synthesis of deoxyribonucleic acid Biochimica et biophysica acta 1956 21 1 197 198 10.1016/0006-3002(56)90127-5 13363894

[B13] Goulian M Kornberg A Enzymatic synthesis of DNA. 23. Synthesis of circular replicative form of phage phi-X174 DNA Proc Natl Acad Sci USA 1967 58 4 1723 1730 10.1073/pnas.58.4.1723 PMC223986 4868446

[B14] Goulian M Kornberg A Sinsheimer RL Enzymatic synthesis of DNA, XXIV. Synthesis of infectious phage phi-X174 DNA Proc Natl Acad Sci USA 1967 58 6 2321 2328 10.1073/pnas.58.6.2321 PMC223838 4873588

[B15] Kornberg T Gefter ML DNA synthesis in cell-free extracts of a DNA polymerase-defective mutant Biochem Biophys Res Commun 1970 40 6 1348 1355 10.1016/0006-291x(70)90014-8 4933688

[B16] Kornberg T Gefter ML Purification and DNA synthesis in cell-free extracts: properties of DNA polymerase II Proc Natl Acad Sci USA 1971 68 4 761 764 10.1073/pnas.68.4.761 PMC389037 4927672

[B17] Kornberg T Gefter ML Deoxyribonucleic acid synthesis in cell-free extracts. IV. Purification and catalytic properties of deoxyribonucleic acid polymerase III J Biol Chem 1972 247 17 5369 5375 4560196

[B18] George J Devoret R Radman M Indirect ultraviolet-reactivation of phage lambda Proc Natl Acad Sci USA 1974 71 1 144 147 10.1073/pnas.71.1.144 PMC387953 4589889

[B19] Lemontt JF Mutants of yeast defective in mutation induced by ultraviolet light Genetics 1971 68 1 21 33 10.1093/genetics/68.1.21 PMC1212581 17248528

[B20] Kato T Shinoura Y Isolation and characterization of mutants of Escherichia coli deficient in induction of mutations by ultraviolet light Mol Gen Genet 1977 156 2 121 131 10.1007/BF00283484 340898

[B21] Lawrence CW Das G Christensen RB REV7, a new gene concerned with UV mutagenesis in yeast Mol Gen Genet 1985 200 1 80 85 10.1007/BF00383316 3897794

[B22] Bonner CA Hays S McEntee K Goodman MF DNA polymerase II is encoded by the DNA damage-inducible dinA gene of Escherichia coli Proc Natl Acad Sci USA 1990 87 19 7663 7667 10.1073/pnas.87.19.7663 PMC54808 2217198

[B23] Iwasaki H Nakata A Walker GC Shinagawa H The Escherichia coli polB gene, which encodes DNA polymerase II, is regulated by the SOS system J Bacteriol 1990 172 11 6268 6273 10.1128/jb.172.11.6268-6273.1990 PMC526809 2228959

[B24] Kenyon CJ Walker GC DNA-damaging agents stimulate gene expression at specific loci in Escherichia coli Proc Natl Acad Sci USA 1980 77 5 2819 2823 10.1073/pnas.77.5.2819 PMC349496 6771759

[B25] Wagner J Gruz P Kim SR Yamada M Matsui K Fuchs RP Nohmi T The dinB gene encodes a novel E. coli DNA polymerase, DNA pol IV, involved in mutagenesis Molecular cell 1999 4 2 281 286 10.1016/s1097-2765(00)80376-7 10488344

[B26] Tang M Shen X Frank EG O'Donnell M Woodgate R Goodman MF UmuD'(2)C is an error-prone DNA polymerase, Escherichia coli pol V Proc Natl Acad Sci USA 1999 96 16 8919 8924 10.1073/pnas.96.16.8919 PMC17708 10430871

[B27] Reuven NB Arad G Maor-Shoshani A Livneh Z The mutagenesis protein UmuC is a DNA polymerase activated by UmuD', RecA, and SSB and is specialized for translesion replication J Biol Chem 1999 274 45 31763 31766 10.1074/jbc.274.45.31763 10542196

[B28] Tang M Pham P Shen X Taylor JS O'Donnell M Woodgate R Goodman MF Roles of E. coli DNA polymerases IV and V in lesion-targeted and untargeted SOS mutagenesis Nature 2000 404 6781 1014 1018 10.1038/35010020 10801133

[B29] McDonald JP Levine AS Woodgate R The Saccharomyces cerevisiae RAD30 gene, a homologue of Escherichia coli dinB and umuC, is DNA damage inducible and functions in a novel error-free postreplication repair mechanism Genetics 1997 147 4 1557 1568 10.1093/genetics/147.4.1557 PMC1208331 9409821

[B30] Sharief FS Vojta PJ Ropp PA Copeland WC Cloning and chromosomal mapping of the human DNA polymerase theta (POLQ), the eighth human DNA polymerase Genomics 1999 59 1 90 96 10.1006/geno.1999.5843 10395804

[B31] Gerlach VL Aravind L Gotway G Schultz RA Koonin EV Friedberg EC Human and mouse homologs of Escherichia coli DinB (DNA polymerase IV), members of the UmuC/DinB superfamily Proc Natl Acad Sci USA 1999 96 21 11922 11927 10.1073/pnas.96.21.11922 PMC18388 10518552

[B32] McDonald JP Rapic-Otrin V Epstein JA Broughton BC Wang X Lehmann AR Wolgemuth DJ Woodgate R Novel human and mouse homologs of Saccharomyces cerevisiae DNA polymerase eta Genomics 1999 60 1 20 30 10.1006/geno.1999.5906 10458907

[B33] Aoufouchi S Flatter E Dahan A Faili A Bertocci B Storck S Delbos F Cocea L Gupta N Weill JC Two novel human and mouse DNA polymerases of the polX family Nucleic acids research 2000 28 18 3684 3693 10.1093/nar/28.18.3684 PMC110747 10982892

[B34] Dominguez O Ruiz JF Lain de Lera T Garcia-Diaz M Gonzalez MA Kirchhoff T Martinez AC Bernad A Blanco L DNA polymerase mu (Pol mu), homologous to TdT, could act as a DNA mutator in eukaryotic cells The EMBO journal 2000 19 7 1731 1742 10.1093/emboj/19.7.1731 PMC310241 10747040

[B35] Garcia-Diaz M Dominguez O Lopez-Fernandez LA de Lera LT Saniger ML Ruiz JF Parraga M Garcia-Ortiz MJ Kirchhoff T del Mazo J DNA polymerase lambda (Pol lambda), a novel eukaryotic DNA polymerase with a potential role in meiosis J Mol Biol 2000 301 4 851 867 10.1006/jmbi.2000.4005 10966791

[B36] Marini F Kim N Schuffert A Wood RD POLN, a nuclear PolA family DNA polymerase homologous to the DNA cross-link sensitivity protein Mus308 J Biol Chem 2003 278 34 32014 32019 10.1074/jbc.M305646200 12794064

[B37] Nelson JR Lawrence CW Hinkle DC Thymine-thymine dimer bypass by yeast DNA polymerase zeta Science 1996 272 5268 1646 1649 10.1126/science.272.5268.1646 8658138

[B38] Nelson JR Lawrence CW Hinkle DC Deoxycytidyl transferase activity of yeast REV1 protein Nature 1996 382 6593 729 731 10.1038/382729a0 8751446

[B39] Lawrence CW Cellular roles of DNA polymerase zeta and Rev1 protein DNA repair 2002 1 6 425 435 10.1016/s1568-7864(02)00038-1 12509231

[B40] Lin W Xin H Zhang Y Wu X Yuan F Wang Z The human REV1 gene codes for a DNA template-dependent dCMP transferase Nucleic acids research 1999 27 22 4468 4475 10.1093/nar/27.22.4468 PMC148731 10536157

[B41] Kannouche P Stary A Xeroderma pigmentosum variant and error-prone DNA polymerases Biochimie 2003 85 11 1123 1132 10.1016/j.biochi.2003.10.009 14726018

[B42] Maher VM Ouellette LM Curren RD McCormick JJ Frequency of ultraviolet light-induced mutations is higher in xeroderma pigmentosum variant cells than in normal human cells Nature 1976 261 5561 593 595 10.1038/261593a0 934300

[B43] Johnson RE Washington MT Haracska L Prakash S Prakash L Eukaryotic polymerases iota and zeta act sequentially to bypass DNA lesions Nature 2000 406 6799 1015 1019 10.1038/35023030 10984059

[B44] Washington MT Minko IG Johnson RE Wolfle WT Harris TM Lloyd RS Prakash S Prakash L Efficient and error-free replication past a minor-groove DNA adduct by the sequential action of human DNA polymerases iota and kappa Mol Cell Biol 2004 24 13 5687 5693 10.1128/MCB.24.13.5687-5693.2004 PMC480884 15199127

[B45] Ogi T Shinkai Y Tanaka K Ohmori H Polkappa protects mammalian cells against the lethal and mutagenic effects of benzo[a]pyrene Proc Natl Acad Sci USA 2002 99 24 15548 15553 10.1073/pnas.222377899 PMC137754 12432099

[B46] Arana ME Seki M Wood RD Rogozin IB Kunkel TA Low-fidelity DNA synthesis by human DNA polymerase theta Nucleic acids research 2008 36 11 3847 3856 10.1093/nar/gkn310 PMC2441791 18503084

[B47] Masuda K Ouchida R Takeuchi A Saito T Koseki H Kawamura K Tagawa M Tokuhisa T Azuma T J OW DNA polymerase theta contributes to the generation of C/G mutations during somatic hypermutation of Ig genes Proc Natl Acad Sci USA 2005 102 39 13986 13991 10.1073/pnas.0505636102 PMC1236561 16172387

[B48] Zan H Shima N Xu Z Al-Qahtani A Evinger Iii AJ Zhong Y Schimenti JC Casali P The translesion DNA polymerase theta plays a dominant role in immunoglobulin gene somatic hypermutation The EMBO journal 2005 24 21 3757 3769 10.1038/sj.emboj.7600833 PMC1276717 16222339

[B49] Bertocci B De Smet A Weill JC Reynaud CA Nonoverlapping functions of DNA polymerases mu, lambda, and terminal deoxynucleotidyltransferase during immunoglobulin V(D)J recombination in vivo Immunity 2006 25 1 31 41 10.1016/j.immuni.2006.04.013 16860755

[B50] Bertocci B De Smet A Berek C Weill JC Reynaud CA Immunoglobulin kappa light chain gene rearrangement is impaired in mice deficient for DNA polymerase mu Immunity 2003 19 2 203 211 10.1016/s1074-7613(03)00203-6 12932354

[B51] Takata K Shimizu T Iwai S Wood RD Human DNA polymerase N (POLN) is a low fidelity enzyme capable of error-free bypass of 5S-thymine glycol J Biol Chem 2006 281 33 23445 23455 10.1074/jbc.M604317200 16787914

[B52] Stallons LJ McGregor WG Translesion synthesis polymerases in the prevention and promotion of carcinogenesis J Nucleic Acids 2010 2010 10.4061/2010/643857 PMC2945679 20936171

[B53] Boudsocq F Kokoska RJ Plosky BS Vaisman A Ling H Kunkel TA Yang W Woodgate R Investigating the role of the little finger domain of Y-family DNA polymerases in low fidelity synthesis and translesion replication J Biol Chem 2004 279 31 32932 32940 10.1074/jbc.M405249200 15155753

[B54] Fortune JM Stith CM Kissling GE Burgers PM Kunkel TA RPA and PCNA suppress formation of large deletion errors by yeast DNA polymerase delta Nucleic acids research 2006 34 16 4335 4341 10.1093/nar/gkl403 PMC1636344 16936322

[B55] Kunkel TA DNA replication fidelity J Biol Chem 2004 279 17 16895 16898 10.1074/jbc.R400006200 14988392

[B56] McCulloch SD Kunkel TA The fidelity of DNA synthesis by eukaryotic replicative and translesion synthesis polymerases Cell Res 2008 18 1 148 161 10.1038/cr.2008.4 PMC3639319 18166979

[B57] Gibbs PE McDonald J Woodgate R Lawrence CW The relative roles in vivo of Saccharomyces cerevisiae Pol eta, Pol zeta, Rev1 protein and Pol32 in the bypass and mutation induction of an abasic site, T-T (6-4) photoadduct and T-T cis-syn cyclobutane dimer Genetics 2005 169 2 575 582 10.1534/genetics.104.034611 PMC1449107 15520252

[B58] Washington MT Johnson RE Prakash S Prakash L Accuracy of thymine-thymine dimer bypass by Saccharomyces cerevisiae DNA polymerase eta Proc Natl Acad Sci USA 2000 97 7 3094 3099 10.1073/pnas.050491997 PMC16198 10725365

[B59] Cordonnier AM Fuchs RP Replication of damaged DNA: molecular defect in xeroderma pigmentosum variant cells Mutation research 1999 435 2 111 119 10.1016/s0921-8777(99)00047-6 10556591

[B60] Yamada A Masutani C Iwai S Hanaoka F Complementation of defective translesion synthesis and UV light sensitivity in xeroderma pigmentosum variant cells by human and mouse DNA polymerase eta Nucleic acids research 2000 28 13 2473 2480 10.1093/nar/28.13.2473 PMC102698 10871396

[B61] Roush AA Suarez M Friedberg EC Radman M Siede W Deletion of the Saccharomyces cerevisiae gene RAD30 encoding an Escherichia coli DinB homolog confers UV radiation sensitivity and altered mutability Mol Gen Genet 1998 257 6 686 692 10.1007/s004380050698 9604893

[B62] Seki M Masutani C Yang LW Schuffert A Iwai S Bahar I Wood RD High-efficiency bypass of DNA damage by human DNA polymerase Q The EMBO journal 2004 23 22 4484 4494 10.1038/sj.emboj.7600424 PMC526458 15496986

[B63] Yoshimura M Kohzaki M Nakamura J Asagoshi K Sonoda E Hou E Prasad R Wilson SH Tano K Yasui A Vertebrate POLQ and POLbeta cooperate in base excision repair of oxidative DNA damage Molecular cell 2006 24 1 115 125 10.1016/j.molcel.2006.07.032 PMC1868411 17018297

[B64] Shachar S Ziv O Avkin S Adar S Wittschieben J Reissner T Chaney S Friedberg EC Wang Z Carell T Two-polymerase mechanisms dictate error-free and error-prone translesion DNA synthesis in mammals The EMBO journal 2009 28 4 383 393 10.1038/emboj.2008.281 PMC2646147 19153606

[B65] Ohmori H Friedberg EC Fuchs RP Goodman MF Hanaoka F Hinkle D Kunkel TA Lawrence CW Livneh Z Nohmi T The Y-family of DNA polymerases Molecular cell 2001 8 1 7 8 10.1016/s1097-2765(01)00278-7 11515498

[B66] Gibbs PE McGregor WG Maher VM Nisson P Lawrence CW A human homolog of the Saccharomyces cerevisiae REV3 gene, which encodes the catalytic subunit of DNA polymerase zeta Proc Natl Acad Sci USA 1998 95 12 6876 6880 10.1073/pnas.95.12.6876 PMC22668 9618506

[B67] Lin W Wu X Wang Z A full-length cDNA of hREV3 is predicted to encode DNA polymerase zeta for damage-induced mutagenesis in humans Mutation research 1999 433 2 89 98 10.1016/s0921-8777(98)00065-2 10102035

[B68] Seki M Marini F Wood RD POLQ (Pol theta), a DNA polymerase and DNA-dependent ATPase in human cells Nucleic acids research 2003 31 21 6117 6126 10.1093/nar/gkg814 PMC275456 14576298

[B69] Prasad R Longley MJ Sharief FS Hou EW Copeland WC Wilson SH Human DNA polymerase theta possesses 5'-dRP lyase activity and functions in single-nucleotide base excision repair in vitro Nucleic acids research 2009 37 6 1868 1877 10.1093/nar/gkp035 PMC2665223 19188258

[B70] Seki M Wood RD DNA polymerase theta (POLQ) can extend from mismatches and from bases opposite a (6-4) photoproduct DNA repair 2008 7 1 119 127 10.1016/j.dnarep.2007.08.005 PMC2185714 17920341

[B71] Maga G Shevelev I Ramadan K Spadari S Hubscher U DNA polymerase theta purified from human cells is a high-fidelity enzyme J Mol Biol 2002 319 2 359 369 10.1016/S0022-2836(02)00325-X 12051913

[B72] Shima N Munroe RJ Schimenti JC The mouse genomic instability mutation chaos1 is an allele of Polq that exhibits genetic interaction with Atm Mol Cell Biol 2004 24 23 10381 10389 10.1128/MCB.24.23.10381-10389.2004 PMC529050 15542845

[B73] Shima N Hartford SA Duffy T Wilson LA Schimenti KJ Schimenti JC Phenotype-based identification of mouse chromosome instability mutants Genetics 2003 163 3 1031 1040 10.1093/genetics/163.3.1031 PMC1462482 12663541

[B74] Ukai A Maruyama T Mochizuki S Ouchida R Masuda K Kawamura K Tagawa M Kinoshita K Sakamoto A Tokuhisa T Role of DNA polymerase theta in tolerance of endogenous and exogenous DNA damage in mouse B cells Genes Cells 2006 11 2 111 121 10.1111/j.1365-2443.2006.00922.x 16436048

[B75] Shivapurkar N Sood S Wistuba II Virmani AK Maitra A Milchgrub S Minna JD Gazdar AF Multiple regions of chromosome 4 demonstrating allelic losses in breast carcinomas Cancer Res 1999 59 15 3576 3580 10446964

[B76] Zietlow L Smith LA Bessho M Bessho T Evidence for the involvement of human DNA polymerase N in the repair of DNA interstrand cross-links Biochemistry 2009 48 49 11817 11824 10.1021/bi9015346 PMC2790558 19908865

[B77] Moldovan GL Madhavan MV Mirchandani KD McCaffrey RM Vinciguerra P D'Andrea AD DNA polymerase POLN participates in cross-link repair and homologous recombination Mol Cell Biol 2010 30 4 1088 1096 10.1128/MCB.01124-09 PMC2815579 19995904

[B78] Kohzaki M Nishihara K Hirota K Sonoda E Yoshimura M Ekino S Butler JE Watanabe M Halazonetis TD Takeda S DNA polymerases nu and theta are required for efficient immunoglobulin V gene diversification in chicken The Journal of cell biology 2010 189 7 1117 1127 10.1083/jcb.200912012 PMC2894443 20584917

[B79] Morrison A Christensen RB Alley J Beck AK Bernstine EG Lemontt JF Lawrence CW REV3, a Saccharomyces cerevisiae gene whose function is required for induced mutagenesis, is predicted to encode a nonessential DNA polymerase J Bacteriol 1989 171 10 5659 5667 10.1128/jb.171.10.5659-5667.1989 PMC210411 2676986

[B80] Braithwaite DK Ito J Compilation, alignment, and phylogenetic relationships of DNA polymerases Nucleic acids research 1993 21 4 787 802 10.1093/nar/21.4.787 PMC309208 8451181

[B81] Murakumo Y Roth T Ishii H Rasio D Numata S Croce CM Fishel R A human REV7 homolog that interacts with the polymerase zeta catalytic subunit hREV3 and the spindle assembly checkpoint protein hMAD2 J Biol Chem 2000 275 6 4391 4397 10.1074/jbc.275.6.4391 10660610

[B82] Murakumo Y The property of DNA polymerase zeta: REV7 is a putative protein involved in translesion DNA synthesis and cell cycle control Mutation research 2002 510 1-2 37 44 10.1016/s0027-5107(02)00250-6 12459441

[B83] Morelli C Mungall AJ Negrini M Barbanti-Brodano G Croce CM Alternative splicing, genomic structure, and fine chromosome localization of REV3L Cytogenet Cell Genet 1998 83 1-2 18 20 10.1159/000015157 9925914

[B84] Van Sloun PP Romeijn RJ Eeken JC Molecular cloning, expression and chromosomal localisation of the mouse Rev3l gene, encoding the catalytic subunit of polymerase zeta Mutation research 1999 433 2 109 116 10.1016/s0921-8777(98)00067-6 10102037

[B85] Morelli C Karayianni E Magnanini C Mungall AJ Thorland E Negrini M Smith DI Barbanti-Brodano G Cloning and characterization of the common fragile site FRA6F harboring a replicative senescence gene and frequently deleted in human tumors Oncogene 2002 21 47 7266 7276 10.1038/sj.onc.1205573 12370818

[B86] Ogawara D Muroya T Yamauchi K Iwamoto TA Yagi Y Yamashita Y Waga S Akiyama M Maki H Near-full-length REV3L appears to be a scarce maternal factor in Xenopus laevis eggs that changes qualitatively in early embryonic development DNA repair 9 1 90 95 10.1016/j.dnarep.2009.10.004 19896909

[B87] J OW Kajiwara K Kawamura K Kimura M Miyagishima H Koseki H Tagawa M An essential role for REV3 in mammalian cell survival: absence of REV3 induces p53-independent embryonic death Biochem Biophys Res Commun 2002 293 3 1132 1137 10.1016/S0006-291X(02)00341-8 12051777

[B88] Esposito G Godindagger I Klein U Yaspo ML Cumano A Rajewsky K Disruption of the Rev3l-encoded catalytic subunit of polymerase zeta in mice results in early embryonic lethality Curr Biol 2000 10 19 1221 1224 10.1016/s0960-9822(00)00726-0 11050393

[B89] Wittschieben J Shivji MK Lalani E Jacobs MA Marini F Gearhart PJ Rosewell I Stamp G Wood RD Disruption of the developmentally regulated Rev3l gene causes embryonic lethality Curr Biol 2000 10 19 1217 1220 10.1016/s0960-9822(00)00725-9 11050392

[B90] Bemark M Khamlichi AA Davies SL Neuberger MS Disruption of mouse polymerase zeta (Rev3) leads to embryonic lethality and impairs blastocyst development in vitro Curr Biol 2000 10 19 1213 1216 10.1016/s0960-9822(00)00724-7 11050391

[B91] Holway AH Kim SH La Volpe A Michael WM Checkpoint silencing during the DNA damage response in Caenorhabditis elegans embryos The Journal of cell biology 2006 172 7 999 1008 10.1083/jcb.200512136 PMC2063758 16549501

[B92] Wittschieben JP Reshmi SC Gollin SM Wood RD Loss of DNA polymerase zeta causes chromosomal instability in mammalian cells Cancer Res 2006 66 1 134 142 10.1158/0008-5472.CAN-05-2982 16397225

[B93] Zander L Bemark M Immortalized mouse cell lines that lack a functional Rev3 gene are hypersensitive to UV irradiation and cisplatin treatment DNA repair 2004 3 7 743 752 10.1016/j.dnarep.2004.03.031 15177183

[B94] Krieg AJ Hammond EM Giaccia AJ Functional analysis of p53 binding under differential stresses Mol Cell Biol 2006 26 19 7030 7045 10.1128/MCB.00322-06 PMC1592883 16980608

[B95] Wu F Lin X Okuda T Howell SB DNA polymerase zeta regulates cisplatin cytotoxicity, mutagenicity, and the rate of development of cisplatin resistance Cancer Res 2004 64 21 8029 8035 10.1158/0008-5472.CAN-03-3942 15520212

[B96] Rajpal DK Wu X Wang Z Alteration of ultraviolet-induced mutagenesis in yeast through molecular modulation of the REV3 and REV7 gene expression Mutation research 2000 461 2 133 143 10.1016/s0921-8777(00)00047-1 11018586

[B97] Brondello JM Pillaire MJ Rodriguez C Gourraud PA Selves J Cazaux C Piette J Novel evidences for a tumor suppressor role of Rev3, the catalytic subunit of Pol zeta Oncogene 2008 27 47 6093 6101 10.1038/onc.2008.212 18622427

[B98] Matsuoka S Ballif BA Smogorzewska A McDonald ER Hurov KE Luo J Bakalarski CE Zhao Z Solimini N Lerenthal Y ATM and ATR substrate analysis reveals extensive protein networks responsive to DNA damage Science 2007 316 5828 1160 1166 10.1126/science.1140321 17525332

[B99] Wittschieben JP Patil V Glushets V Robinson LJ Kusewitt DF Wood RD Loss of DNA Polymerase {zeta} Enhances Spontaneous Tumorigenesis Cancer Res 10.1158/0008-5472.CAN-09-4267 PMC3008348 20215524

[B100] Li Z Zhang H McManus TP McCormick JJ Lawrence CW Maher VM hREV3 is essential for error-prone translesion synthesis past UV or benzo[a]pyrene diol epoxide-induced DNA lesions in human fibroblasts Mutation research 2002 510 1-2 71 80 10.1016/s0027-5107(02)00253-1 12459444

[B101] Diaz M Watson NB Turkington G Verkoczy LK Klinman NR McGregor WG Decreased frequency and highly aberrant spectrum of ultraviolet-induced mutations in the hprt gene of mouse fibroblasts expressing antisense RNA to DNA polymerase zeta Mol Cancer Res 2003 1 11 836 847 14517346

[B102] Xie K Doles J Hemann MT Walker GC Error-prone translesion synthesis mediates acquired chemoresistance Proc Natl Acad Sci USA 2010 107 48 20792 20797 10.1073/pnas.1011412107 PMC2996453 21068378

[B103] Doles J Oliver TG Cameron ER Hsu G Jacks T Walker GC Hemann MT Suppression of Rev3, the catalytic subunit of Pol{zeta}, sensitizes drug-resistant lung tumors to chemotherapy Proc Natl Acad Sci USA 2010 107 48 20786 20791 10.1073/pnas.1011409107 PMC2996428 21068376

[B104] Zhang N Liu X Li L Legerski R Double-strand breaks induce homologous recombinational repair of interstrand cross-links via cooperation of MSH2, ERCC1-XPF, REV3, and the Fanconi anemia pathway DNA repair 2007 6 11 1670 1678 10.1016/j.dnarep.2007.06.002 PMC2586762 17669695

[B105] Hicks JK Chute CL Paulsen MT Ragland RL Howlett NG Gueranger Q Glover TW Canman CE Differential roles for DNA polymerases eta, zeta, and REV1 in lesion bypass of intrastrand versus interstrand DNA cross-links Mol Cell Biol 2010 30 5 1217 1230 10.1128/MCB.00993-09 PMC2820889 20028736

[B106] Schenten D Kracker S Esposito G Franco S Klein U Murphy M Alt FW Rajewsky K Pol zeta ablation in B cells impairs the germinal center reaction, class switch recombination, DNA break repair, and genome stability J Exp Med 2009 206 2 477 490 10.1084/jem.20080669 PMC2646585 19204108

[B107] Murakumo Y Ogura Y Ishii H Numata S Ichihara M Croce CM Fishel R Takahashi M Interactions in the error-prone postreplication repair proteins hREV1, hREV3, and hREV7 J Biol Chem 2001 276 38 35644 35651 10.1074/jbc.M102051200 11485998

[B108] Aravind L Koonin EV The HORMA domain: a common structural denominator in mitotic checkpoints, chromosome synapsis and DNA repair Trends Biochem Sci 1998 23 8 284 286 10.1016/s0968-0004(98)01257-2 9757827

[B109] Pfleger CM Salic A Lee E Kirschner MW Inhibition of Cdh1-APC by the MAD2-related protein MAD2L2: a novel mechanism for regulating Cdh1 Genes Dev 2001 15 14 1759 1764 10.1101/gad.897901 PMC312740 11459825

[B110] Iwai H Kim M Yoshikawa Y Ashida H Ogawa M Fujita Y Muller D Kirikae T Jackson PK Kotani S A bacterial effector targets Mad2L2, an APC inhibitor, to modulate host cell cycling Cell 2007 130 4 611 623 10.1016/j.cell.2007.06.043 17719540

[B111] McBride OW Kozak CA Wilson SH Mapping of the gene for DNA polymerase beta to mouse chromosome 8 Cytogenet Cell Genet 1990 53 2-3 108 111 10.1159/000132906 1973375

[B112] Kumar A Widen SG Williams KR Kedar P Karpel RL Wilson SH Studies of the domain structure of mammalian DNA polymerase beta. Identification of a discrete template binding domain J Biol Chem 1990 265 4 2124 2131 2404980

[B113] Casas-Finet JR Kumar A Morris G Wilson SH Karpel RL Spectroscopic studies of the structural domains of mammalian DNA beta-polymerase J Biol Chem 1991 266 29 19618 19625 1918069

[B114] Pelletier H Sawaya MR Kumar A Wilson SH Kraut J Structures of ternary complexes of rat DNA polymerase beta, a DNA template-primer, and ddCTP Science 1994 264 5167 1891 1903 7516580

[B115] Beard WA Shock DD Yang XP DeLauder SF Wilson SH Loss of DNA polymerase beta stacking interactions with templating purines, but not pyrimidines, alters catalytic efficiency and fidelity J Biol Chem 2002 277 10 8235 8242 10.1074/jbc.M107286200 11756435

[B116] Singhal RK Wilson SH Short gap-filling synthesis by DNA polymerase beta is processive J Biol Chem 1993 268 21 15906 15911 8340415

[B117] Prasad R Beard WA Wilson SH Studies of gapped DNA substrate binding by mammalian DNA polymerase beta. Dependence on 5'-phosphate group J Biol Chem 1994 269 27 18096 18101 8027071

[B118] Matsumoto Y Kim K Excision of deoxyribose phosphate residues by DNA polymerase beta during DNA repair Science 1995 269 5224 699 702 10.1126/science.7624801 7624801

[B119] Dianov GL Prasad R Wilson SH Bohr VA Role of DNA polymerase beta in the excision step of long patch mammalian base excision repair J Biol Chem 1999 274 20 13741 13743 10.1074/jbc.274.20.13741 10318775

[B120] Biade S Sobol RW Wilson SH Matsumoto Y Impairment of proliferating cell nuclear antigen-dependent apurinic/apyrimidinic site repair on linear DNA J Biol Chem 1998 273 2 898 902 10.1074/jbc.273.2.898 9422747

[B121] Sobol RW Horton JK Kuhn R Gu H Singhal RK Prasad R Rajewsky K Wilson SH Requirement of mammalian DNA polymerase-beta in base-excision repair Nature 1996 379 6561 183 186 10.1038/379183a0 8538772

[B122] Hoffmann JS Pillaire MJ Maga G Podust V Hubscher U Villani G DNA polymerase beta bypasses in vitro a single d(GpG)-cisplatin adduct placed on codon 13 of the HRAS gene Proc Natl Acad Sci USA 1995 92 12 5356 5360 10.1073/pnas.92.12.5356 PMC41693 7777511

[B123] Esposito G Texido G Betz UA Gu H Muller W Klein U Rajewsky K Mice reconstituted with DNA polymerase beta-deficient fetal liver cells are able to mount a T cell-dependent immune response and mutate their Ig genes normally Proc Natl Acad Sci USA 2000 97 3 1166 1171 10.1073/pnas.97.3.1166 PMC15557 10655502

[B124] Gu H Marth JD Orban PC Mossmann H Rajewsky K Deletion of a DNA polymerase beta gene segment in T cells using cell type-specific gene targeting Science 1994 265 5168 103 106 10.1126/science.8016642 8016642

[B125] Horton JK Srivastava DK Zmudzka BZ Wilson SH Strategic down-regulation of DNA polymerase beta by antisense RNA sensitizes mammalian cells to specific DNA damaging agents Nucleic acids research 1995 23 19 3810 3815 10.1093/nar/23.19.3810 PMC307295 7479021

[B126] Horton JK Baker A Berg BJ Sobol RW Wilson SH Involvement of DNA polymerase beta in protection against the cytotoxicity of oxidative DNA damage DNA repair 2002 1 4 317 333 10.1016/s1568-7864(02)00008-3 12509250

[B127] Sobol RW Watson DE Nakamura J Yakes FM Hou E Horton JK Ladapo J Van Houten B Swenberg JA Tindall KR Mutations associated with base excision repair deficiency and methylation-induced genotoxic stress Proc Natl Acad Sci USA 2002 99 10 6860 6865 10.1073/pnas.092662499 PMC124494 11983862

[B128] Sobol RW Prasad R Evenski A Baker A Yang XP Horton JK Wilson SH The lyase activity of the DNA repair protein beta-polymerase protects from DNA-damage-induced cytotoxicity Nature 2000 405 6788 807 810 10.1038/35015598 10866204

[B129] Bergoglio V Pillaire MJ Lacroix-Triki M Raynaud-Messina B Canitrot Y Bieth A Gares M Wright M Delsol G Loeb LA Deregulated DNA polymerase beta induces chromosome instability and tumorigenesis Cancer Res 2002 62 12 3511 3514 12067997

[B130] Canitrot Y Laurent G Astarie-Dequeker C Bordier C Cazaux C Hoffmann JS Enhanced expression and activity of DNA polymerase beta in chronic myelogenous leukemia Anticancer Res 2006 26 1B 523 525 16739313

[B131] Garcia-Diaz M Bebenek K Sabariegos R Dominguez O Rodriguez J Kirchhoff T Garcia-Palomero E Picher AJ Juarez R Ruiz JF DNA polymerase lambda, a novel DNA repair enzyme in human cells J Biol Chem 2002 277 15 13184 13191 10.1074/jbc.M111601200 11821417

[B132] Yu X Chini CC He M Mer G Chen J The BRCT domain is a phospho-protein binding domain Science 2003 302 5645 639 642 10.1126/science.1088753 14576433

[B133] Ramadan K Maga G Shevelev IV Villani G Blanco L Hubscher U Human DNA polymerase lambda possesses terminal deoxyribonucleotidyl transferase activity and can elongate RNA primers: implications for novel functions J Mol Biol 2003 328 1 63 72 10.1016/s0022-2836(03)00265-1 12683997

[B134] Bebenek K Garcia-Diaz M Blanco L Kunkel TA The frameshift infidelity of human DNA polymerase lambda. Implications for function J Biol Chem 2003 278 36 34685 34690 10.1074/jbc.M305705200 12829698

[B135] Picher AJ Garcia-Diaz M Bebenek K Pedersen LC Kunkel TA Blanco L Promiscuous mismatch extension by human DNA polymerase lambda Nucleic acids research 2006 34 11 3259 3266 10.1093/nar/gkl377 PMC1904104 16807316

[B136] Ma Y Lu H Tippin B Goodman MF Shimazaki N Koiwai O Hsieh CL Schwarz K Lieber MR A biochemically defined system for mammalian nonhomologous DNA end joining Molecular cell 2004 16 5 701 713 10.1016/j.molcel.2004.11.017 15574326

[B137] Terrados G Capp JP Canitrot Y Garcia-Diaz M Bebenek K Kirchhoff T Villanueva A Boudsocq F Bergoglio V Cazaux C Characterization of a natural mutator variant of human DNA polymerase lambda which promotes chromosomal instability by compromising NHEJ PLoS One 2009 4 10 e7290 10.1371/journal.pone.0007290 PMC2751832 19806195

[B138] Garcia-Diaz M Bebenek K Kunkel TA Blanco L Identification of an intrinsic 5'-deoxyribose-5-phosphate lyase activity in human DNA polymerase lambda: a possible role in base excision repair J Biol Chem 2001 276 37 34659 34663 10.1074/jbc.M106336200 11457865

[B139] Zhang Y Wu X Yuan F Xie Z Wang Z Highly frequent frameshift DNA synthesis by human DNA polymerase mu Mol Cell Biol 2001 21 23 7995 8006 10.1128/MCB.21.23.7995-8006.2001 PMC99967 11689691

[B140] Gu J Lu H Tippin B Shimazaki N Goodman MF Lieber MR XRCC4:DNA ligase IV can ligate incompatible DNA ends and can ligate across gaps The EMBO journal 2007 26 4 1010 1023 10.1038/sj.emboj.7601559 PMC1852838 17290226

[B141] Nair DT Johnson RE Prakash S Prakash L Aggarwal AK Replication by human DNA polymerase-iota occurs by Hoogsteen base-pairing Nature 2004 430 6997 377 380 10.1038/nature02692 15254543

[B142] Uljon SN Johnson RE Edwards TA Prakash S Prakash L Aggarwal AK Crystal structure of the catalytic core of human DNA polymerase kappa Structure 2004 12 8 1395 1404 10.1016/j.str.2004.05.011 15296733

[B143] Silvian LF Toth EA Pham P Goodman MF Ellenberger T Crystal structure of a DinB family error-prone DNA polymerase from Sulfolobus solfataricus Nat Struct Biol 2001 8 11 984 989 10.1038/nsb1101-984 11685247

[B144] Trincao J Johnson RE Escalante CR Prakash S Prakash L Aggarwal AK Structure of the catalytic core of S. cerevisiae DNA polymerase eta: implications for translesion DNA synthesis Molecular cell 2001 8 2 417 426 10.1016/s1097-2765(01)00306-9 11545743

[B145] Bienko M Green CM Crosetto N Rudolf F Zapart G Coull B Kannouche P Wider G Peter M Lehmann AR Ubiquitin-binding domains in Y-family polymerases regulate translesion synthesis Science 2005 310 5755 1821 1824 10.1126/science.1120615 16357261

[B146] Ohashi E Murakumo Y Kanjo N Akagi J Masutani C Hanaoka F Ohmori H Interaction of hREV1 with three human Y-family DNA polymerases Genes Cells 2004 9 6 523 531 10.1111/j.1356-9597.2004.00747.x 15189446

[B147] Lawrence CW Cellular functions of DNA polymerase zeta and Rev1 protein Adv Protein Chem 2004 69 167 203 10.1016/S0065-3233(04)69006-1 15588843

[B148] Guo C Tang TS Bienko M Parker JL Bielen AB Sonoda E Takeda S Ulrich HD Dikic I Friedberg EC Ubiquitin-binding motifs in REV1 protein are required for its role in the tolerance of DNA damage Mol Cell Biol 2006 26 23 8892 8900 10.1128/MCB.01118-06 PMC1636806 16982685

[B149] Tissier A Kannouche P Reck MP Lehmann AR Fuchs RP Cordonnier A Co-localization in replication foci and interaction of human Y-family members, DNA polymerase pol eta and REVl protein DNA repair 2004 3 11 1503 1514 10.1016/j.dnarep.2004.06.015 15380106

[B150] Guo C Sonoda E Tang TS Parker JL Bielen AB Takeda S Ulrich HD Friedberg EC REV1 protein interacts with PCNA: significance of the REV1 BRCT domain in vitro and in vivo Molecular cell 2006 23 2 265 271 10.1016/j.molcel.2006.05.038 16857592

[B151] Simpson LJ Sale JE Rev1 is essential for DNA damage tolerance and non-templated immunoglobulin gene mutation in a vertebrate cell line The EMBO journal 2003 22 7 1654 1664 10.1093/emboj/cdg161 PMC152905 12660171

[B152] Sarkies P Reams C Simpson LJ Sale JE Epigenetic Instability due to Defective Replication of Structured DNA Molecular cell 2010 40 5 703 713 10.1016/j.molcel.2010.11.009 PMC3145961 21145480

[B153] Masutani C Kusumoto R Yamada A Dohmae N Yokoi M Yuasa M Araki M Iwai S Takio K Hanaoka F The XPV (xeroderma pigmentosum variant) gene encodes human DNA polymerase eta Nature 1999 399 6737 700 704 10.1038/21447 10385124

[B154] Acharya N Yoon JH Gali H Unk I Haracska L Johnson RE Hurwitz J Prakash L Prakash S Roles of PCNA-binding and ubiquitin-binding domains in human DNA polymerase eta in translesion DNA synthesis Proc Natl Acad Sci USA 2008 105 46 17724 17729 10.1073/pnas.0809844105 PMC2584706 19001268

[B155] Yagi Y Ogawara D Iwai S Hanaoka F Akiyama M Maki H DNA polymerases eta and kappa are responsible for error-free translesion DNA synthesis activity over a cis-syn thymine dimer in Xenopus laevis oocyte extracts DNA repair 2005 4 11 1252 1269 10.1016/j.dnarep.2005.06.010 16055392

[B156] Wang Y Woodgate R McManus TP Mead S McCormick JJ Maher VM Evidence that in xeroderma pigmentosum variant cells, which lack DNA polymerase eta, DNA polymerase iota causes the very high frequency and unique spectrum of UV-induced mutations Cancer Res 2007 67 7 3018 3026 10.1158/0008-5472.CAN-06-3073 17409408

[B157] Jansen JG Tsaalbi-Shtylik A Langerak P Calleja F Meijers CM Jacobs H de Wind N The BRCT domain of mammalian Rev1 is involved in regulating DNA translesion synthesis Nucleic acids research 2005 33 1 356 365 10.1093/nar/gki189 PMC546167 15653636

[B158] Delbos F De Smet A Faili A Aoufouchi S Weill JC Reynaud CA Contribution of DNA polymerase eta to immunoglobulin gene hypermutation in the mouse J Exp Med 2005 201 8 1191 1196 10.1084/jem.20050292 PMC2213152 15824086

[B159] Ogi T Kannouche P Lehmann AR Localisation of human Y-family DNA polymerase kappa: relationship to PCNA foci J Cell Sci 2005 118 Pt 1 129 136 10.1242/jcs.01603 15601657

[B160] Ziv O Geacintov N Nakajima S Yasui A Livneh Z DNA polymerase zeta cooperates with polymerases kappa and iota in translesion DNA synthesis across pyrimidine photodimers in cells from XPV patients Proc Natl Acad Sci USA 2009 106 28 11552 11557 10.1073/pnas.0812548106 PMC2710681 19564618

[B161] Ogi T Limsirichaikul S Overmeer RM Volker M Takenaka K Cloney R Nakazawa Y Niimi A Miki Y Jaspers NG Three DNA polymerases, recruited by different mechanisms, carry out NER repair synthesis in human cells Molecular cell 2010 37 5 714 727 10.1016/j.molcel.2010.02.009 20227374

[B162] Bavoux C Leopoldino AM Bergoglio V J OW Ogi T Bieth A Judde JG Pena SD Poupon MF Helleday T Up-regulation of the error-prone DNA polymerase {kappa} promotes pleiotropic genetic alterations and tumorigenesis Cancer Res 2005 65 1 325 330 15665310

[B163] Bebenek K Tissier A Frank EG McDonald JP Prasad R Wilson SH Woodgate R Kunkel TA 5'-Deoxyribose phosphate lyase activity of human DNA polymerase iota in vitro Science 2001 291 5511 2156 2159 10.1126/science.1058386 11251121

[B164] Petta TB Nakajima S Zlatanou A Despras E Couve-Privat S Ishchenko A Sarasin A Yasui A Kannouche P Human DNA polymerase iota protects cells against oxidative stress The EMBO journal 2008 27 21 2883 2895 10.1038/emboj.2008.210 PMC2580789 18923427

[B165] Kannouche P Fernandez de Henestrosa AR Coull B Vidal AE Gray C Zicha D Woodgate R Lehmann AR Localization of DNA polymerases eta and iota to the replication machinery is tightly co-ordinated in human cells The EMBO journal 2003 22 5 1223 1233 10.1093/emboj/cdf618 PMC150329 12606586

[B166] Kannouche PL Wing J Lehmann AR Interaction of human DNA polymerase eta with monoubiquitinated PCNA: a possible mechanism for the polymerase switch in response to DNA damage Molecular cell 2004 14 4 491 500 10.1016/s1097-2765(04)00259-x 15149598

[B167] Watanabe K Tateishi S Kawasuji M Tsurimoto T Inoue H Yamaizumi M Rad18 guides poleta to replication stalling sites through physical interaction and PCNA monoubiquitination The EMBO journal 2004 23 19 3886 3896 10.1038/sj.emboj.7600383 PMC522788 15359278

[B168] Andersen PL Xu F Xiao W Eukaryotic DNA damage tolerance and translesion synthesis through covalent modifications of PCNA Cell Res 2008 18 1 162 173 10.1038/cr.2007.114 18157158

[B169] Chang DJ Cimprich KA DNA damage tolerance: when it's OK to make mistakes Nat Chem Biol 2009 5 2 82 90 10.1038/nchembio.139 PMC2663399 19148176

[B170] Unk I Hajdu I Fatyol K Szakal B Blastyak A Bermudez V Hurwitz J Prakash L Prakash S Haracska L Human SHPRH is a ubiquitin ligase for Mms2-Ubc13-dependent polyubiquitylation of proliferating cell nuclear antigen Proc Natl Acad Sci USA 2006 103 48 18107 18112 10.1073/pnas.0608595103 PMC1838714 17108083

[B171] Unk I Hajdu I Fatyol K Hurwitz J Yoon JH Prakash L Prakash S Haracska L Human HLTF functions as a ubiquitin ligase for proliferating cell nuclear antigen polyubiquitination Proc Natl Acad Sci USA 2008 105 10 3768 3773 10.1073/pnas.0800563105 PMC2268824 18316726

[B172] Ulrich HD Deubiquitinating PCNA: a downside to DNA damage tolerance Nat Cell Biol 2006 8 4 303 305 10.1038/ncb0406-303 16607265

[B173] Huang TT Nijman SM Mirchandani KD Galardy PJ Cohn MA Haas W Gygi SP Ploegh HL Bernards R D'Andrea AD Regulation of monoubiquitinated PCNA by DUB autocleavage Nat Cell Biol 2006 8 4 339 347 10.1038/ncb1378 16531995

[B174] Wang W Emergence of a DNA-damage response network consisting of Fanconi anaemia and BRCA proteins Nat Rev Genet 2007 8 10 735 748 10.1038/nrg2159 17768402

[B175] Hicks JK Chute CL Paulsen MT Ragland RL Howlett NG Gueranger Q Glover TW Canman CE Differential roles for DNA polymerases eta, zeta, and REV1 in lesion bypass of intrastrand versus interstrand DNA cross-links Mol Cell Biol 30 5 1217 1230 10.1128/MCB.00993-09 PMC2820889 20028736

[B176] Mirchandani KD McCaffrey RM D'Andrea AD The Fanconi anemia core complex is required for efficient point mutagenesis and Rev1 foci assembly DNA repair 2008 7 6 902 911 10.1016/j.dnarep.2008.03.001 PMC2715953 18448394

[B177] Sabbioneda S Minesinger BK Giannattasio M Plevani P Muzi-Falconi M Jinks-Robertson S The 9-1-1 checkpoint clamp physically interacts with polzeta and is partially required for spontaneous polzeta-dependent mutagenesis in Saccharomyces cerevisiae J Biol Chem 2005 280 46 38657 38665 10.1074/jbc.M507638200 16169844

[B178] Friedberg EC Lehmann AR Fuchs RP Trading places: how do DNA polymerases switch during translesion DNA synthesis? Molecular cell 2005 18 5 499 505 10.1016/j.molcel.2005.03.032 15916957

[B179] Lopes M Foiani M Sogo JM Multiple mechanisms control chromosome integrity after replication fork uncoupling and restart at irreparable UV lesions Molecular cell 2006 21 1 15 27 10.1016/j.molcel.2005.11.015 16387650

[B180] Waters LS Walker GC The critical mutagenic translesion DNA polymerase Rev1 is highly expressed during G(2)/M phase rather than S phase Proc Natl Acad Sci USA 2006 103 24 8971 8976 10.1073/pnas.0510167103 PMC1482550 16751278

[B181] Jansen JG Tsaalbi-Shtylik A Hendriks G Verspuy J Gali H Haracska L de Wind N Mammalian polymerase zeta is essential for post-replication repair of UV-induced DNA lesions DNA repair 2009 8 12 1444 1451 10.1016/j.dnarep.2009.09.006 19783229

[B182] Edmunds CE Simpson LJ Sale JE PCNA ubiquitination and REV1 define temporally distinct mechanisms for controlling translesion synthesis in the avian cell line DT40 Molecular cell 2008 30 4 519 529 10.1016/j.molcel.2008.03.024 18498753

[B183] Fung H Demple B Distinct roles of Ape1 protein in the repair of DNA damage induced by ionizing radiation or bleomycin J Biol Chem 2011 286 7 4968 4977 10.1074/jbc.M110.146498 PMC3037609 21081487

[B184] Blanca G Villani G Shevelev I Ramadan K Spadari S Hubscher U Maga G Human DNA polymerases lambda and beta show different efficiencies of translesion DNA synthesis past abasic sites and alternative mechanisms for frameshift generation Biochemistry 2004 43 36 11605 11615 10.1021/bi049050x 15350147

[B185] Efrati E Tocco G Eritja R Wilson SH Goodman MF Abasic translesion synthesis by DNA polymerase beta violates the "A-rule". Novel types of nucleotide incorporation by human DNA polymerase beta at an abasic lesion in different sequence contexts J Biol Chem 1997 272 4 2559 2569 10.1074/jbc.272.4.2559 8999973

[B186] Masutani C Kusumoto R Iwai S Hanaoka F Mechanisms of accurate translesion synthesis by human DNA polymerase eta The EMBO journal 2000 19 12 3100 3109 10.1093/emboj/19.12.3100 PMC203367 10856253

[B187] Ramadan K Shevelev IV Maga G Hubscher U DNA polymerase lambda from calf thymus preferentially replicates damaged DNA J Biol Chem 2002 277 21 18454 18458 10.1074/jbc.M200421200 11886860

[B188] Maga G Villani G Ramadan K Shevelev I Tanguy Le Gac N Blanco L Blanca G Spadari S Hubscher U Human DNA polymerase lambda functionally and physically interacts with proliferating cell nuclear antigen in normal and translesion DNA synthesis J Biol Chem 2002 277 50 48434 48440 10.1074/jbc.M206889200 12368291

[B189] Ohashi E Ogi T Kusumoto R Iwai S Masutani C Hanaoka F Ohmori H Error-prone bypass of certain DNA lesions by the human DNA polymerase kappa Genes Dev 2000 14 13 1589 1594 PMC316741 10887153

[B190] Zhang Y Wu X Guo D Rechkoblit O Taylor JS Geacintov NE Wang Z Lesion bypass activities of human DNA polymerase mu J Biol Chem 2002 277 46 44582 44587 10.1074/jbc.M207297200 12228225

[B191] Choi JY Lim S Kim EJ Jo A Guengerich FP Translesion synthesis across abasic lesions by human B-family and Y-family DNA polymerases alpha, delta, eta, iota, kappa, and REV1 J Mol Biol 2010 404 1 34 44 10.1016/j.jmb.2010.09.015 PMC3018708 20888339

[B192] Croteau DL Bohr VA Repair of oxidative damage to nuclear and mitochondrial DNA in mammalian cells J Biol Chem 1997 272 41 25409 25412 10.1074/jbc.272.41.25409 9325246

[B193] Lindahl T Instability and decay of the primary structure of DNA Nature 1993 362 6422 709 715 10.1038/362709a0 8469282

[B194] Olinski R Zastawny T Budzbon J Skokowski J Zegarski W Dizdaroglu M DNA base modifications in chromatin of human cancerous tissues FEBS Lett 1992 309 2 193 198 10.1016/0014-5793(92)81093-2 1324197

[B195] Avkin S Livneh Z Efficiency, specificity and DNA polymerase-dependence of translesion replication across the oxidative DNA lesion 8-oxoguanine in human cells Mutation research 2002 510 1-2 81 90 10.1016/s0027-5107(02)00254-3 12459445

[B196] Irimia A Eoff RL Guengerich FP Egli M Structural and functional elucidation of the mechanism promoting error-prone synthesis by human DNA polymerase kappa opposite the 7, 8-dihydro-8-oxo-2'-deoxyguanosine adduct J Biol Chem 2009 284 33 22467 22480 10.1074/jbc.M109.003905 PMC2755968 19542228

[B197] Zhang Y Yuan F Wu X Taylor JS Wang Z Response of human DNA polymerase iota to DNA lesions Nucleic acids research 2001 29 4 928 935 10.1093/nar/29.4.928 PMC29608 11160925

[B198] Maga G Villani G Crespan E Wimmer U Ferrari E Bertocci B Hubscher U 8-oxo-guanine bypass by human DNA polymerases in the presence of auxiliary proteins Nature 2007 447 7144 606 608 10.1038/nature05843 17507928

[B199] Maga G Crespan E Wimmer U van Loon B Amoroso A Mondello C Belgiovine C Ferrari E Locatelli G Villani G Replication protein A and proliferating cell nuclear antigen coordinate DNA polymerase selection in 8-oxo-guanine repair Proc Natl Acad Sci USA 2008 105 52 20689 20694 10.1073/pnas.0811241106 PMC2634905 19104052

[B200] Belousova EA Maga G Fan Y Kubareva EA Romanova EA Lebedeva NA Oretskaya TS Lavrik OI DNA polymerases beta and lambda bypass thymine glycol in gapped DNA structures Biochemistry 2010 49 22 4695 4704 10.1021/bi901792c 20423048

[B201] Yoon JH Bhatia G Prakash S Prakash L Error-free replicative bypass of thymine glycol by the combined action of DNA polymerases kappa and zeta in human cells Proc Natl Acad Sci USA 2010 107 32 14116 14121 10.1073/pnas.1007795107 PMC2922520 20660785

[B202] Lindahl T Wood RD Quality control by DNA repair Science 1999 286 5446 1897 1905 10.1126/science.286.5446.1897 10583946

[B203] Hoeijmakers JH DNA damage, aging, and cancer N Engl J Med 2009 361 15 1475 1485 10.1056/NEJMra0804615 19812404

[B204] Kim JK Patel D Choi BS Contrasting structural impacts induced by cis-syn cyclobutane dimer and (6-4) adduct in DNA duplex decamers: implication in mutagenesis and repair activity Photochem Photobiol 1995 62 1 44 50 10.1111/j.1751-1097.1995.tb05236.x 7638271

[B205] Yoon JH Prakash L Prakash S Error-free replicative bypass of (6-4) photoproducts by DNA polymerase zeta in mouse and human cells Genes Dev 2010 24 2 123 128 10.1101/gad.1872810 PMC2807347 20080950

[B206] Park H Zhang K Ren Y Nadji S Sinha N Taylor JS Kang C Crystal structure of a DNA decamer containing a cis-syn thymine dimer Proc Natl Acad Sci USA 2002 99 25 15965 15970 10.1073/pnas.242422699 PMC138548 12456887

[B207] Johnson RE Washington MT Prakash S Prakash L Fidelity of human DNA polymerase eta J Biol Chem 2000 275 11 7447 7450 10.1074/jbc.275.11.7447 10713043

[B208] McCulloch SD Kokoska RJ Masutani C Iwai S Hanaoka F Kunkel TA Preferential cis-syn thymine dimer bypass by DNA polymerase eta occurs with biased fidelity Nature 2004 428 6978 97 100 10.1038/nature02352 14999287

[B209] Hendel A Ziv O Gueranger Q Geacintov N Livneh Z Reduced efficiency and increased mutagenicity of translesion DNA synthesis across a TT cyclobutane pyrimidine dimer, but not a TT 6-4 photoproduct, in human cells lacking DNA polymerase eta DNA repair 2008 7 10 1636 1646 10.1016/j.dnarep.2008.06.008 PMC2656611 18634905

[B210] Denissenko MF Pao A Tang M Pfeifer GP Preferential formation of benzo[a]pyrene adducts at lung cancer mutational hotspots in P53 Science 1996 274 5286 430 432 10.1126/science.274.5286.430 8832894

[B211] Avkin S Goldsmith M Velasco-Miguel S Geacintov N Friedberg EC Livneh Z Quantitative analysis of translesion DNA synthesis across a benzo[a]pyrene-guanine adduct in mammalian cells: the role of DNA polymerase kappa J Biol Chem 2004 279 51 53298 53305 10.1074/jbc.M409155200 15475561

[B212] Vaisman A Chaney SG The efficiency and fidelity of translesion synthesis past cisplatin and oxaliplatin GpG adducts by human DNA polymerase beta J Biol Chem 2000 275 17 13017 13025 10.1074/jbc.275.17.13017 10777605

[B213] Vaisman A Masutani C Hanaoka F Chaney SG Efficient translesion replication past oxaliplatin and cisplatin GpG adducts by human DNA polymerase eta Biochemistry 2000 39 16 4575 4580 10.1021/bi000130k 10769112

[B214] Vaisman A Lim SE Patrick SM Copeland WC Hinkle DC Turchi JJ Chaney SG Effect of DNA polymerases and high mobility group protein 1 on the carrier ligand specificity for translesion synthesis past platinum-DNA adducts Biochemistry 1999 38 34 11026 11039 10.1021/bi9909187 10460158

[B215] Havener JM Nick McElhinny SA Bassett E Gauger M Ramsden DA Chaney SG Translesion synthesis past platinum DNA adducts by human DNA polymerase mu Biochemistry 2003 42 6 1777 1788 10.1021/bi0270079 12578393

[B216] Deans AJ West SC DNA interstrand crosslink repair and cancer Nat Rev Cancer 2011 11 7 467 480 10.1038/nrc3088 PMC3560328 21701511

[B217] Stone MP Cho YJ Huang H Kim HY Kozekov ID Kozekova A Wang H Minko IG Lloyd RS Harris TM Interstrand DNA cross-links induced by alpha, beta-unsaturated aldehydes derived from lipid peroxidation and environmental sources Acc Chem Res 2008 41 7 793 804 10.1021/ar700246x PMC2785109 18500830

[B218] Minko IG Harbut MB Kozekov ID Kozekova A Jakobs PM Olson SB Moses RE Harris TM Rizzo CJ Lloyd RS Role for DNA polymerase kappa in the processing of N2-N2-guanine interstrand cross-links J Biol Chem 2008 283 25 17075 17082 10.1074/jbc.M801238200 PMC2427349 18434313

[B219] Albertella MR Green CM Lehmann AR O'Connor MJ A role for polymerase eta in the cellular tolerance to cisplatin-induced damage Cancer Res 2005 65 21 9799 9806 10.1158/0008-5472.CAN-05-1095 16267001

[B220] Chen YW Cleaver JE Hanaoka F Chang CF Chou KM A novel role of DNA polymerase eta in modulating cellular sensitivity to chemotherapeutic agents Mol Cancer Res 2006 4 4 257 265 10.1158/1541-7786.MCR-05-0118 16603639

[B221] Shen X Jun S O'Neal LE Sonoda E Bemark M Sale JE Li L REV3 and REV1 play major roles in recombination-independent repair of DNA interstrand cross-links mediated by monoubiquitinated proliferating cell nuclear antigen (PCNA) J Biol Chem 2006 281 20 13869 13872 10.1074/jbc.C600071200 16571727

[B222] Raschle M Knipscheer P Enoiu M Angelov T Sun J Griffith JD Ellenberger TE Scharer OD Walter JC Mechanism of replication-coupled DNA interstrand crosslink repair Cell 2008 134 6 969 980 10.1016/j.cell.2008.08.030 PMC2748255 18805090

[B223] Masuda Y Ohmae M Masuda K Kamiya K Structure and enzymatic properties of a stable complex of the human REV1 and REV7 proteins J Biol Chem 2003 278 14 12356 12360 10.1074/jbc.M211765200 12529368

[B224] Hlavin EM Smeaton MB Noronha AM Wilds CJ Miller PS Cross-link structure affects replication-independent DNA interstrand cross-link repair in mammalian cells Biochemistry 2010 49 18 3977 3988 10.1021/bi902169q PMC2864352 20373772

[B225] Quah SK von Borstel RC Hastings PJ The origin of spontaneous mutation in Saccharomyces cerevisiae Genetics 1980 96 4 819 839 10.1093/genetics/96.4.819 PMC1219303 7021317

[B226] Starcevic D Dalal S Sweasy JB Is there a link between DNA polymerase beta and cancer? Cell Cycle 2004 3 8 998 1001 15280658

[B227] Lee GH Matsushita H Genetic linkage between Pol iota deficiency and increased susceptibility to lung tumors in mice Cancer Sci 2005 96 5 256 259 10.1111/j.1349-7006.2005.00042.x PMC11158430 15904465

[B228] Wang M Devereux TR Vikis HG McCulloch SD Holliday W Anna C Wang Y Bebenek K Kunkel TA Guan K Pol iota is a candidate for the mouse pulmonary adenoma resistance 2 locus, a major modifier of chemically induced lung neoplasia Cancer Res 2004 64 6 1924 1931 10.1158/0008-5472.can-03-3080 15026325

[B229] Sakiyama T Kohno T Mimaki S Ohta T Yanagitani N Sobue T Kunitoh H Saito R Shimizu K Hirama C Association of amino acid substitution polymorphisms in DNA repair genes TP53, POLI, REV1 and LIG4 with lung cancer risk Int J Cancer 2005 114 5 730 737 10.1002/ijc.20790 15609317

[B230] Albertella MR Lau A O'Connor MJ The overexpression of specialized DNA polymerases in cancer DNA repair 2005 4 5 583 593 10.1016/j.dnarep.2005.01.005 15811630

[B231] J OW Kawamura K Tada Y Ohmori H Kimura H Sakiyama S Tagawa M DNA polymerase kappa, implicated in spontaneous and DNA damage-induced mutagenesis, is overexpressed in lung cancer Cancer Res 2001 61 14 5366 5369 11454676

[B232] Yang J Chen Z Liu Y Hickey RJ Malkas LH Altered DNA polymerase iota expression in breast cancer cells leads to a reduction in DNA replication fidelity and a higher rate of mutagenesis Cancer Res 2004 64 16 5597 5607 10.1158/0008-5472.CAN-04-0603 15313897

[B233] Lemee F Bergoglio V Fernandez-Vidal A Machado-Silva A Pillaire MJ Bieth A Gentil C Baker L Martin AL Leduc C DNA polymerase theta up-regulation is associated with poor survival in breast cancer, perturbs DNA replication, and promotes genetic instability Proc Natl Acad Sci USA 2010 107 30 13390 13395 10.1073/pnas.0910759107 PMC2922118 20624954

[B234] Bartkova J Horejsi Z Koed K Kramer A Tort F Zieger K Guldberg P Sehested M Nesland JM Lukas C DNA damage response as a candidate anti-cancer barrier in early human tumorigenesis Nature 2005 434 7035 864 870 10.1038/nature03482 15829956

[B235] Fong PC Boss DS Yap TA Tutt A Wu P Mergui-Roelvink M Mortimer P Swaisland H Lau A O'Connor MJ Inhibition of poly(ADP-ribose) polymerase in tumors from BRCA mutation carriers N Engl J Med 2009 361 2 123 134 10.1056/NEJMoa0900212 19553641

[B236] Martin SA McCabe N Mullarkey M Cummins R Burgess DJ Nakabeppu Y Oka S Kay E Lord CJ Ashworth A DNA polymerases as potential therapeutic targets for cancers deficient in the DNA mismatch repair proteins MSH2 or MLH1 Cancer cell 2010 17 3 235 248 10.1016/j.ccr.2009.12.046 PMC2845806 20227038

[B237] Knobel PA Kotov IN Felley-Bosco E Stahel RA Marti TM Inhibition of *REV3 *expression induces persistent DNA damage and growth arrest in cancer cells Neoplasia (New York, NY 2011 13 10 961 970 10.1593/neo.11828 PMC3201572 22028621

[B238] Wang Z DNA damage-induced mutagenesis: a novel target for cancer prevention Mol Interv 2001 1 5 269 281 14993366

[B239] Northam MR Robinson HA Kochenova OV Shcherbakova PV Participation of DNA polymerase zeta in replication of undamaged DNA in Saccharomyces cerevisiae Genetics 2010 184 1 27 42 10.1534/genetics.109.107482 PMC2815923 19841096

[B240] Izuta S Inhibition of DNA polymerase eta by oxetanocin derivatives Nucleic Acids Symp Ser (Oxf) 2006 50 269 270 10.1093/nass/nrl134 17150921

[B241] Mizushina Y Kamisuki S Kasai N Ishidoh T Shimazaki N Takemura M Asahara H Linn S Yoshida S Koiwai O Petasiphenol: a DNA polymerase lambda inhibitor Biochemistry 2002 41 49 14463 14471 10.1021/bi020476q 12463744

[B242] Matsubara K Mori M Mizushina Y Petasiphenol which inhibits DNA polymerase lambda activity is an inhibitor of in vitro angiogenesis Oncol Rep 2004 11 2 447 451 14719082

[B243] Maga G Hubscher U Repair and translesion DNA polymerases as anticancer drug targets Anticancer Agents Med Chem 2008 8 4 431 447 10.2174/187152008784220348 18473728

[B244] Degregori J Evolved tumor suppression: why are we so good at not getting cancer? Cancer Res 2011 71 11 3739 3744 10.1158/0008-5472.CAN-11-0342 PMC3677553 21610109

[B245] Waters LS Minesinger BK Wiltrout ME D'Souza S Woodruff RV Walker GC Eukaryotic translesion polymerases and their roles and regulation in DNA damage tolerance Microbiol Mol Biol Rev 2009 73 1 134 154 10.1128/MMBR.00034-08 PMC2650891 19258535

[B246] Lange SS Takata K Wood RD DNA polymerases and cancer Nat Rev Cancer 2011 11 2 96 110 10.1038/nrc2998 PMC3739438 21258395

[B247] Livneh Z Ziv O Shachar S Multiple two-polymerase mechanisms in mammalian translesion DNA synthesis Cell Cycle 2010 9 4 729 735 10.4161/cc.9.4.10727 20139724

